# ﻿Morphometric and phylogenetic analysis of a commercial fish *Leiognathusequula* (Teleostei, Leiognathidae)

**DOI:** 10.3897/zookeys.1219.130546

**Published:** 2024-12-04

**Authors:** Jiajie Chen, Xiaodong Wang, Sheng Zeng, Wei Tian, Deyuan Yang, Jinqing Ye, Junsheng Zhong, Chaopeng Jiang

**Affiliations:** 1 Shanghai Universities Key Laboratory of Marine Animal Taxonomy and Evolution, Shanghai Ocean University, 201306, Shanghai, China; 2 East China Sea Fisheries Research Institute, Fisheries Science of Chinese Academy, 200090, Shanghai, China; 3 College of the Environment and Ecology, Xiamen University, 361102, Xiamen, China; 4 National Taiwan Ocean University, Keelung, 202301, Taiwan; 5 National Marine Environment Monitoring Center, 116023, Dalian, China; 6 College of Marine Living Resource Sciences and Management, Shanghai Ocean University, 201306, Shanghai, China

**Keywords:** Leiognathidae, *Leiognathusequula*, mitochondrial genome, phylogenetic tree, South China Sea

## Abstract

The complete mitochondrial genome and phylogenetic analysis for *Leiognathusequula* from the South China Sea was performed using shallow genome skimming. For accurate species identification and redescription, morphometric and meristic characters were examined and compared with previous descriptions. To facilitate the identification of species and to enable comparison with the mitochondrial genome phylogeny, molecular comparisons were conducted using three mitochondrial genes: large ribosomal RNA (*16S* rRNA), cytochrome c oxidase subunit 1 (*COX1*), and NADH dehydrogenase (*ND5*). The mitogenome (16 398 bp) comprised 38 mitochondrial genes, similar to most bony fishes: 13 protein-coding genes (PCGs), 2 rRNA and 22 transfer RNA genes, and 1 non-coding region. The complete mitogenome comprised 30.7% A, 24.2% T, 15.0% G, and 30.1% C. The A+T content (54.9%) was higher than the G+C content (45.1%). All PCGs started with the typical ATG codon, except *COX1*, which started with GTG. Seven PCGs ended with incomplete stop codons (TA or T). The Ka/Ks ratios of all PCGs were < 1, indicating purifying selection. The phylogenies of Leiognathidae, both based on the amino acid sequences of the 13 PCGs and the single genes *16S* RNA and *COX1*, were monophyletic with high nodal support (> 75%). *L.brevirostris* (NC 026232) is believed to be a *Nuchequula* species, whereas *L.ruconius* (NC 057225) is not classified under *Leiognathus* in the NCBI database. The phylogenetic trees divided the Leiognathidae family into three clades. The mitogenome phylogeny suggested that the Leiognathidae and Chaetodontidae clades are sister groups. These findings provide important genetic data for population genetics research and a phylogenetic analysis of Leiognathidae.

## ﻿Introduction

Leiognathids (family Leiognathidae), commonly known as ponyfishes or slipmouths, are characterized by their highly protractile mouths that extend dorsorostrally, rostrally, or ventrorostrally. The relationship of Leiognathidae—an ex-perciform—with the order Perciformes is currently under debate. In the new taxonomical classiﬁcation, Leiognathidae were reclassified from Perciformes to Chaetodontiformes (Betancur-R et al. 2017; Schoch et al. 2020). However, osteological evidence suggests that Leiognathidae should be classified as Acanthuriformes (Gill and Leis 2019; Gill and Michalski 2020).

All members of the family Leiognathidae possess a distinctive circumesophageal light organ that houses bioluminescent bacteria belonging to the genus *Photobacterium*. Males possess a larger light organ and associated features that intensify light during sexual displays for photic communication (Sparks et al. 2005). Many species of leiognathid fish exhibit strong sexual dimorphism in relation to their light organ system (LOS), except *Aurigequula* and *Leiognathus* species. Leiognathids are difficult to distinguish and identify if the features of the LOS are not considered. This difficulty may be due to the fact that both internal and external characteristics are coservative. As a result, there are several putatively widespread “wastebasket” species, such as *Aurigequulafasciata* (Lacepède, 1803) and *Leiognathusequula* (Forsskål, 1775) (Sparks et al. 2005).

*Leiognathusequula* is a tropical inshore bottom-dwelling panfish with a large, robust, and rhomboid-shaped body (Chakrabarty et al. 2008). It is widely distributed in both the Red Sea and the Indo-West Pacific Ocean (Masuda et al. 1984; Chakrabarty and Sparks 2022). In China, *L.equula* is known to be found from coastal regions of Taiwan (Shen 1993; Chakrabarty et al. 2010; Shen and Wu 2011; Gao et al. 2020) and Taiwan Strait (Chen and Fang 1999; Chen and Yang 2013; Liu et al. 2014) to the South China Sea, including coastal areas of Nansha Islands (Chen et al. 1997; Chen and Ni 2021), Hainan Island (Zheng 1962; Gao et al. 2020; this study), Guangdong (Yan et al. 2021), Beibu Gulf, Guangxi (Liu et al. 2016; Lai and He 2016). *Leiognathusequula* is a senior synonym of *Leiognathusargenteus* Lacepède, 1802, which is the type species of *Leiognathus*. It is significantly larger and more robust than other Leiognathidae species, but it can still be confused with other species due to its conservative morphology (Chen and Yang 2013).

The species is fished commercially in South China; it is one of the most economically important species in Hainan and is often sold together with silver pomfret (*Pampusargenteus*) but at approximately half the price of the latter (Suppl. material 1: fig. S1). However, to date, the species has not received sufficient research attention. Currently, *L.equula* has the conservation status of “Least Concern (LC)” (Larson et al. 2017). In the field of conservation biology, one of the most challenging aspects of species conservation is the effective identification of species. From a commercial perspective, it is of significant interest to identify species for traceability and to enhance the accuracy of their labelling. The mitogenome may prove useful in the design of molecular tools for both objectives.

In recent studies, some species previously classified as *Leiognathus* have been reclassified as belonging to other genera (Ikejima et al. 2004; Sparks and Dunlap 2004; Sparks et al. 2005; Chakrabarty and Sparks 2007, 2008, 2022; Chakrabarty et al. 2008, 2011; Kimura et al. 2008a, 2008b, 2008c; Sparks and Chakrabarty 2015; Suzuki and Kimura 2023). There are currently ten monophyletic genera in Leiognathidae, which is now widely accepted (see Sparks and Chakrabarty 2015). They appear to be almost identical in terms of their morphology, but there are significant genetic differences between them (Seah et al. 2008). Consequently, genetic analyses constitute an invaluable tool for the identification of species (Seishi Kimura, pers. comm.). Nevertheless, previous phylogenetic studies of the family Leiognathidae have been based on a single gene (Chakrabarty et al. 2011; Ikejima et al. 2004; Sparks and Dunlap 2004; Sparks et al. 2005; Dunlap et al. 2007; Seah et al. 2008; Seth and Barik 2021). The complete mitochondrial genome of Leiognathidae species has yet to be the subject of extensive research. The addition of further species data on mitochondrial genomes will enable more accurate delineation of the family phylogeny.

The objective of this paper is to provide a description of the mitochondrial hologeny of *L.equula*, a basal species of Leiognathidae (Ikejima et al. 2004). However, published whole-gene data could cause confusion and errors in phylogenetic analyses if specimens were not reviewed and the most recent taxonomic status studies were not identified. The authors of this study have taken the initiative to present a basis for morphological identification and to demonstrate the reliability of species identification with the results of a single-gene study, thereby enhancing the credibility of the mitochondrial whole-gene data.

To achieve this objective, eight specimens were collected from the South China Sea and identified as *L.equula* following a process of careful documentation and morphometric comparison. To identify the species accurately, we compared the mitochondrial DNA sequence data of 16S rRNA, COX1, and ND5 fragments and compared the results of the phylogenetic relationships among Leiognathidae species. The genetic relationships within Leiognathidae were analyzed by assembling the mitochondrial genome of *L.equula* and selecting one of the three mitochondrial genome datasets for description. The present findings enhance the understanding of the mitochondrial genome features of the Leiognathidae and its taxonomic classification. Furthermore, they provide crucial genetic data for phylogenetic and population genetic studies of the family Leiognathidae.

## ﻿Materials and methods

### ﻿Sample collection and DNA extraction

**Figure 1.**
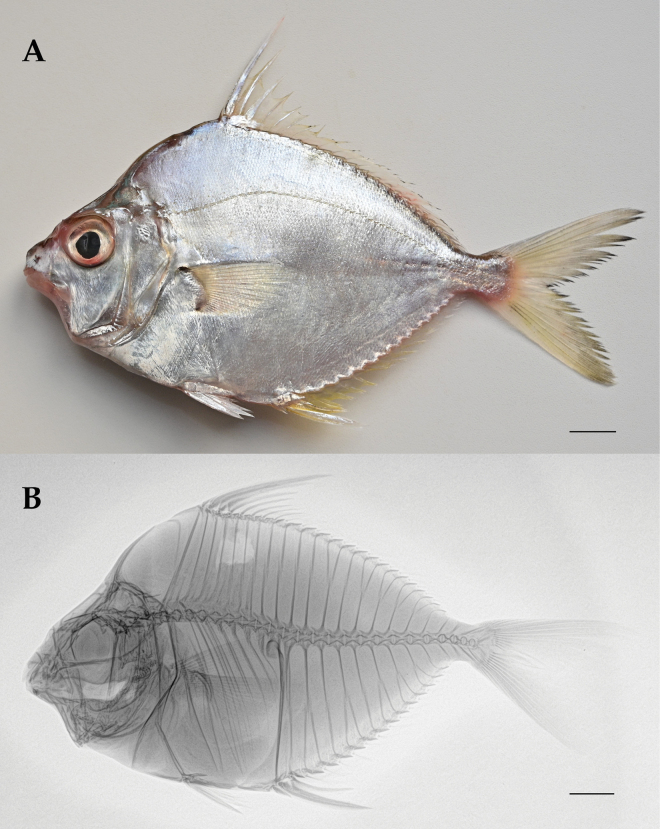
Left lateral view of *L.equula* specimen DHS19056 (SL 105.8 mm) **A** showing color in life (photo by Weiyue Zhang) and **B** radiograph. The whitish area below the dorsal fin indicates where the muscle tissue was sampled. Body depth **A** was measured at the anal-fin origin, and body depth **B** was measured at the dorsal-fin origin. Scale bars: 10 mm.

Eight specimens morphologically identified as *L.equula* were collected from various sources in China, including landing points, fish markets, and onboard commercial and research vessels (Suppl. material 1: table S1, Fig. 1A). They were subsequently stored in 70% ethanol at the East China Sea Fisheries Research Institute, Fisheries Science of Chinese Academy. Before DNA isolation, the surface of the specimens was cleaned with 100% ethanol. Muscle tissue measuring approximately 10 × 10 mm^2^ (Fig. 1B) was collected from below the right lateral dorsal fin of the three of eight specimens (voucher numbers DHS14327, DHS19056, and DHS22490) for subsequent DNA extraction. Whole genomic DNA was extracted using the TIANamp Genomic DNA kit (TIANGEN, Beijing, China).

### ﻿Species identification

Morphological identification was performed following the original description (Niebuhr 1775), in comparison with other published descriptions (Bloch 1795; Lacepède 1802; Cuvier 1829; James 1975; Masuda et al. 1984) and recent publications (Chen and Fang 1999; Kimura and Peristiwady 2000; Carpenter and Niem 2001; Chakrabarty et al. 2008, 2010; Kimura et al. 2009, 2018; Shen and Wu 2011; Chen and Yang 2013; White et al. 2013; Liu et al. 2014; Psomadakis et al. 2015, 2019; Lai and He 2016; Liu et al. 2016; Yan et al. 2021; Gloerfelt-Tarp and Kailola 2022). Counts and measurements were taken following Sparks and Dunlap (2004) and Hubbs and Lagler (2004). All measurements were taken using digital calipers accurate to within 0.01 mm. Radiographs were used to examine osteological features. Standard length and head length are abbreviated as SL and HL, respectively. The scales were examined and counted using cyanine blue (Saruwatari et al. 1997). Scale counts are approximations owing to high intraspecific and interspecific variability, irregular arrangements, and the small size of scales, which makes accurate counting challenging.

The assembled sequence was subjected to BLAST using the NCBI BLAST similarity search tool. Subsequently, the *16S*, *COX1*, and *ND5* sequences from NCBI (Suppl. material 1: tables S2, S3) were selected for alignment with mitochondrial fragments, with the objective of verifying species accuracy. In light of the limited availability of mitogenome data for *Leiognathus*, the *16S rRNA*, *COX1*, and *ND5* sequences were also employed to investigate the phylogenetic position of *L.equula*, with a view to comparing the results with those of phylogenetic analyses of the mitogenome.

### ﻿Mitogenome sequencing and assembly

The DNA library was prepared by the Illumina TruseqTM DNA Sample Preparation Kit (Illumina, San Diego, USA) using the manufacturer’s instructions. The prepared library was sequenced by Novogene Bioinformatics Technology Co., Ltd. (Beijing, China) using the DNBSEQ-T7 platform to generate 150 bp paired-end reads. In total, ~ 5 Gb of raw sequence data were generated for each sample. Data cleaning was performed using Fastp v. 0.23.2 with default parameters (Chen et al. 2018). FastQC v. 0.11.5 (http://www.bioinformatics.babraham.ac.uk/projects/fastqc/) was used to assess the quality of raw data and cleaned data (Suppl. material 1: table S4). The assembly pipeline FastMitoAssembler (https://github.com/suqingdong/FastMitoAssembler) was used to assemble the mitochondrial genome, and then GetOrganelle v. 1.7.6.1 (Jin et al. 2020) and NovoPlasty v. 4.3.1 (Dierckxsens et al. 2017) are two commonly used organelle genome assemblers.

### ﻿Mitogenome annotation and sequence analyses

The mitochondrial genome was annotated using MITOS2 (Donath et al. 2019) and the Mitoz annotation module (Meng et al. 2019). Geneious v. 2021.0.3 was used to check the annotated sequences manually. The base composition and codon usage were calculated, and the relative synonymous codon usage (RSCU) of each protein-coding gene (PCG) was analyzed using PhyloSuite 1.2.3 (Zhang et al. 2020). The following general formulae were applied to estimate A+T skew and G+C skew, respectively: A+T skew = (A% − T%)/(A% + T%) and G+C skew = (G% − C%)/(G% + C%) (Perna and Kocher 1995). To investigate the selective pressure, we calculated the ratios of nonsynonymous and synonymous substitutions (Ka/Ks) in the mitogenomes of all Leiognathidae species using DnaSP 6.0 (Lowe and Chan 2016). tRNA genes were plotted in the ViennaRNA Web Services (http://rna.tbi.univie.ac.at/forna/) according to the secondary structure predicted by tRNAscan-SE 2.0 (Lowe and Chan 2016; Chan et al. 2021) and MITOS Web Server (Bernt et al. 2013) using the vertebrate mitochondrial genetic code. The sequences of the termination-associated sequence (TAS), central conserved sequence blocks (CSB-F, CSB-E, CSB-D, CSB-B, CSB-A), and conserved sequence block domains (CSB-2, CSB-3) in the control region were identified using the Basic Local Alignment Search Tool (BLAST) against the sequences of the reported fish.

### ﻿Phylogenetic analysis

In order to guarantee the greatest possible accuracy in species identification, a random selection of three regions was tested from a sample of eight. The results were consistent across the three sample assemblies, and we selected OR344340 for description. In order to ascertain the phylogenetic position of species within the Leiognathidae family, we reconstructed a phylogeny of the family Leiognathidae using the mitogenome sequences from the GenBank database (https://ncbi.nlm.nih.gov/) for 36 species (accessed 6 February 2024), including *Lagocephalusgloveri* (Abe and Tabeta 1983) and *Amblygobiusphalaena* (Valenciennes, in Cuvier and Valenciennes 1837), which were used as outgroups (Suppl. material 1: table S5). Sequences were downloaded from GenBank (Suppl. material 1: table S5) to establish the database for phylogenetic analysis using PhyloSuite 1.2.3 (Zhang et al. 2020). Next, 13 PCGs of these mitogenomes were extracted, and each coding gene was aligned using the codon alignment mode in Mafft v. 7.313 (Katoh and Standley 2013). Ambiguous regions were removed using Gblocks 0.91 (Castresana 2000), and the best-fit partition models—(GTR+F+I+G4) for maximum likelihood (ML) and (GTR+F+I+G) for Bayesian inference (BI)—were selected by ModelFinder (Kalyaanamoorthy et al. 2017) using the BICc and AICc criterion, respectively.

The ML analysis was performed in IQ-TREE v. 2.2.2 (Nguyen et al. 2015), under the Edge-linked partition model for 200 000 ultrafast bootstraps. The BI analysis was performed in MrBayes v. 3.2.7a (Ronquist et al. 2012), under the partition model (two parallel runs, 2 000 000 generations). Finally, iTOL v. 6 (Letunic and Bork 2021) was used to visualize the ML and BI phylogenetic trees.

## ﻿Results

### ﻿Species description based on morphological identification

Description based on eight specimens ranging 81.33–144.02 mm in standard length (SL; Suppl. material 1: table S6). Counts and proportional measurements are given in Table 1. D VIII 16; A III 14; P 20; V I–6; C 17; pored scales in lateral line 63–70; vertebrae (precaudal 9 + caudal 14) = 23 (Fig. 1).

**Table 1.** Comparison of morphometric and meristic characters of *L.equula* in the present study and previous studies.

Counts and measurements	Present study (*n* = 8)	Sparks and Dunlap (2004) (*n* = 22)	Chakrabarty et al. (2008) (*n* = 7)	Zheng (1962) (*n* = 10)
Standard length (mm)	105.99 (81.33–144.02)	110.6 (69.0–177.8)	92–128.8	60–232
**Counts**				
Dorsal fin rays	VIII 16	VII–VIII 12–17	—	VIII 16
Anal fin rays	III 14	III 14	—	III 14
Pectoral fin rays	20	19	—	20
Pored scales in lateral line	63–70	56–65	50–60	58–67
Vertebrae (precaudal+caudal)	9+14 = 23	9+14 = 23	—	—
**Measurements**				
**As % of SL**				
Head length	33.21 (31.47–35.91)	30.3 (28.6–33.1)	31.8 (29.8–34.5)	28.74–31.65
Body depth A (origin anal fin)	55.61 (52.95–59.56)	56.2 (51.2–60.5)	—	51.81–57.80
Body depth B (origin dorsal fin)	57.08 (54.76–60.38)	55.1 (49.5–58.3)	57.3 (53.9–61.8)	—
Head width (max.)	12.29 (11.52–13.22)	—	16.1 (15.2–17.6)	—
Caudal peduncle length	5.52 (4.66–6.73)	11.1 (9.4–13.7)	8.3 (6.8–10.6)	—
Caudal peduncle depth	7.57 (7.08–8.25)	6.7 (5.9–7.4)	6.9 (6.5–7.1)	—
Caudal peduncle width	4.26 (3.42–5.10)	4.0 (3.3–4.7)	4.2 (3.6–4.7)	—
Pectoral fin length	25.73 (23.20–27.18)	23.9 (21.2–26.2)	22.0 (20.1–24.1)	—
Pelvic fin length	15.64 (12.58–17.24)	15.5 (11.4–16.8)	16.6 (15.0–18.1)	—
Dorsal fin base length	57.79 (56.34–59.70)	56.2 (53.2–59.1)	—	—
Anal fin base length	47.08 (45.05–50.79)	45.6 (42.3–48.8)	—	—
Predorsal length	52.49 (50.22–54.32)	50.8 (48.4–53.3)	52.5 (50.5–54.0)	—
Prepelvic length	41.04 (37.62–44.85)	37.3 (35.1–40.2)	43.1 (37.2–50.5)	—
Preanal length	57.16 (54.71–59.36)	54.1 (50.2–58.5)	58.2 (56.6–60.2)	—
**As % of HL**				
Snout length	36.37 (33.26–40.37)	37.0 (33.7–39.6)	34.9 (33.3–37.3)	26.11–32.79
Head width (max.)	37.05 (35.02–42.00)	50.9 (47.9–54.4)	—	—
Upper jaw length	40.48 (38.26–43.04)	25.4 (23.1–27.6)	38.9 (35.0–41.3)	—
Lower jaw length	36.85 (34.90–38.84)	52.0 (48.0–57.4)	40.0 (25.7–60.0)	—
Interorbital width	32.02 (25.31–38.48)	34.3 (31.7–36.6)	36.8 (25.7–43.5)	—
Orbital diameter	31.12 (28.58–33.54)	37.3 (33.3–41.7)	34.6 (30.0–37.6)	29.67–35.34
Preorbital depth	54.00 (48.60–58.99)	22.7 (19.6–25.9)		—

Body robust and large, laterally compressed, rhomboid and deep. Dorsal profile more convex than ventral profile. Greatest body depth at vertical from dorsal fin origin to abdomen. Dorsal fin origin posterior to pelvic fin origins. Anal fin origin vertical through first dorsal fin ray (Fig. 1B). Dorsal head slightly triangular in shape, enclosed by 2 supraorbital ridges, apposed nuchal spine which exceeds eye diameter (Suppl. material 1: fig. S2); back typically slightly to strongly arched (Fig. 1). Snout truncated; length slightly equal to eye diameter. Gill opening large. Lower preopercular right-angled, margin weakly serrate. Branchiostegals 5, branchiostegal membrane attached along lateral margin of isthmus. Caudal peduncle short and shallow. Vertebral count: 9 precaudal + 14 caudal = 23. Neural and hemal spines of vertebral centrum PU4 expanded and bladelike (Fig. 1B).

Mouth small and terminal, directed slightly downward, forming tube when protruded. Cleft slightly sloping downward. Lower jaw strongly concave, forming 45° angle when mouth closed. Gape horizontal with inferior eye margin. Lips fleshy but thin. Maxilla exposed, through orbital anterior margin.

Eye moderately large, placed high, lower margin above body axis. Preorbital spine with ridge serrated. Adipose eye lid underdeveloped. Interorbital slightly convex. One short spine on anterior superior margin of eye and posterior of nostril. Nostrils above eyes, two on each side. Anterior nasal pore small and round, posterior large and oblong.

Fins: Dorsal fin 1, with eight spines and 16 rays. First dorsal fin spine very short; second dorsal fin spine longest; 80.35 (77.50–86.39) %HL and 25.26 (15.09–27.83) %SL. Third and fourth dorsal fin spine margin anteriorly serrate. Anal fin with three spines and 14 rays. Second anal fin spine longest; 59.06 (51.79–65.10) %HL and 19.61 (16.98–21.43) %SL. The anal fin spine margin anteriorly serrate. Both dorsal and anal fin base anteriorly covered by membranous sheaths. Pectoral fins rounded and wide. Subthoracic ventral fin shorter than pectoral fin; large axillary scale on pelvic fins; spines retract when laid flat. Terminal ventral fin reached anal fin origin in juveniles (DHS14500, DHS22489, DHS22490). Caudal fin forked, tips of both lobes round and blunt.

Squamation: Lateral line slightly arched posteriorly from the pectoral fin base to the caudal peduncle, continuing horizontally along the caudal peduncle. Head and chest asquamate, body cycloid scales. Lateral line slightly curved and complete, includes 63–70 pored scales.

Dentition: Teeth pointed and bristled, with three or four tooth rows on upper and lower jaw, with incurve, banded arrangement. Vomer, palatine, and tongue toothless.

Fresh coloration: Body greyish to silvery, with compact grey-black narrow band on back and sides; a dark brown saddle on caudal peduncle. Axil of pectoral fins grey to black. Pelvic fins white. Margin of soft dorsal fin black; no dark spots. Margin of soft anal fin yellow. Caudal fin yellowish with black margin. Concentration of melanophores on snout side (Suppl. material 1: fig. S2). Large axillary scales on pelvic fins silvery (Fig. 1A).

Coloration in preservative: Body yellowish. Color pattern of fins similar to fresh coloration but with a yellowish tinge.

### ﻿Molecular identification and analysis

Owing to the limited availability of mitochondrial genome data in Leiognathidae, single mitochondrial genes were chosen as the basis for molecular identification. Phylogenetic reconstruction of the family Leiognathidae on the basis of mitochondrial genes *16S* rRNA, *COX1*, and *ND5* was performed using BI and ML methods. The sequences of *L.equula* from different waters clustered together in all three single-gene phylogenetic trees (Suppl. material 1: figs S3–S5), suggesting that the molecular findings are consistent with the morphological results.

The Hongsha (PP551518) sequences clustered with those from the Philippines (AY541653) and Malaysia (EU366341) in the *16S* rRNA BI tree and with those from the Philippines in the *16S* rRNA ML tree (Suppl. material 1: fig. S3). The Yangxi (PP551517) and Changjiang (OR344340) sequences clustered with those from India (MK644023) in the *16S* rRNA BI tree. In both the BI and ML trees based on the *COX1* gene (Suppl. material 1: fig. S4), Hongsha (PP551518) sequences clustered with sequences from India (MK689371) and Taiwan, China (DQ028018) as sister branches. In addition, Yangxi (PP551517) and Changjiang (OR344340) clustered together in the BI tree, while they were sister branches in the ML tree. In both the BI and ML trees based on the *ND5* gene, the Philippines sequence (AB100017) clustered with the Yangxi (PP551517) and Changjiang (OR344340) sequences (Suppl. material 1: fig. S5), respectively. Additionally, Changjiang (OR344340) and Hongsha (PP551518) were sister branches in the BI tree, whereas Yangxi (PP551517) and Hongsha (PP551518) were sister branches in the ML tree.

### ﻿Mitochondrial genomic structure and base composition

**Figure 2.**
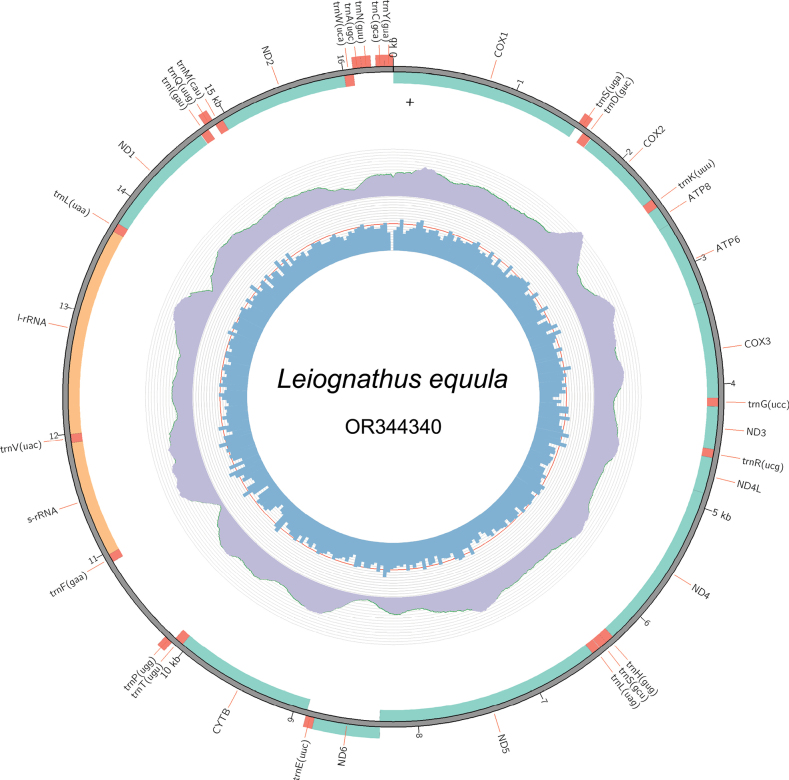
Complete mitogenome of *L.equula*. The middle and innermost circles represent depth distribution and GC content, respectively. The outermost circle shows gene arrangement, with green, orange, and red depicting protein-coding gene fragments, rRNA genes, and tRNA genes, respectively.

The mitochondrial genome of *L.equula* (GenBank accession number: OR344340) was 16,398 bp (Fig. 2), in accordance with other Leiognathidae species (Suppl. material 1: table S5). Similar to the mitogenome of most fish, the *L.equula* mitogenome contained 37 mitochondrial genes (13 typical PCGs, 22 tRNA genes, and 2 rRNA genes) and a control region (Fig. 2, Suppl. material 1: table S7). The ND6 gene and eight tRNA genes (*trnS*[uga], *trnE*[uuc], *trnP*[ugg], *trnQ*[uug], *trnA*[ugc], *trnN*[guu], *trnC*[gca] and *trnY*[gua]) were located on the L chain. The remaining mitochondrial genes were located on the H chain (Fig. 2, Suppl. material 1: table S7). There were six overlapping regions (1–10 bp in size) in the mitogenome. The longest overlapping regions (10 bp) were located between *trnK* (uuu)/ATP8. Furthermore, the mitochondrial genome included 12 gene spacers, which exhibited a size range of 1–40 bp. The longest gap (40 nucleotides) was identified between *trnA* (ugc) and *trnN* (guu) at.

The *L.equula* mitogenome showed a slight bias towards A and T nucleotides, which comprised 54.9% of the total base composition (A = 30.7%, T = 24.2%, G = 15.0%, and C = 30.1%). This was accompanied by a positive A+T skew (0.117) and a negative G+C skew (−0.334) (Suppl. material 1: table S8). Thus, the *L.equula* mitogenome exhibited a clear A+T preference in its base composition, which is consistent with that in other Leiognathidae species (Suppl. material 1: table S5). Compared with the whole genome, the control region had the highest A+T content (approximately 62.8%; Suppl. material 1: table S8), which is a typical feature of the mitochondrial genomes of animals (Zhang and Hewitt 1997; Satoh et al. 2016). In contrast, the first codon position of the PCGs had the lowest A+T content, 49.0% (Suppl. material 1: table S8).

### ﻿Protein-coding genes and codon usage

The total length of PCGs was 11,421 bp, with gene length ranging from 177 bp (*ATP8*) to 1830 bp (*ND5*). In total, 12 PCGs had canonical ATG start codons, whereas the *COX1* gene had a GTG start codon. A complete stop codon was observed in seven PCGs, whereas the remaining six PCGs exhibited an incomplete stop codon (TA or T) at their respective termini—*ATP6* and *COX3* were terminated by TA and *COX2*, CYTB, *ND3*, and *ND4* were terminated by T (Suppl. material 1: table S7). Furthermore, the values of A+T skew and G+C skew for the PCGs were 0.017 and –0.353, respectively, suggesting a higher abundance of A and C nucleotides than that of their respective counterparts (Suppl. material 1: table S8).

**Figure 3.**
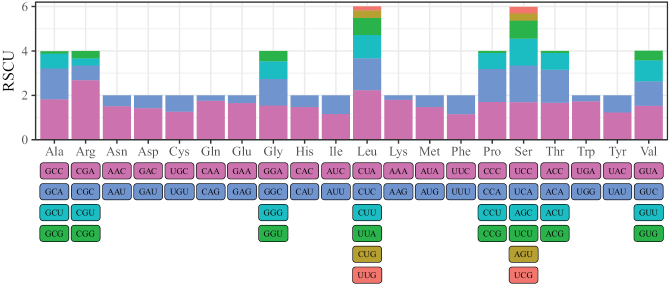
Relative synonymous codon usage (RSCU) of all protein-coding genes in the mitogenome of *L.equula*.

Suppl. material 1: table S9, Fig. 3 provide a summary of the amino acid usage and RSCU values in the PCGs of *L.equula*. In total, 3800 amino acids were encoded in the mitogenome. Of these, leucine (16.84%) and cysteine (0.71%) were the most and least frequently used amino acids, respectively. The six most frequently used codons in *L.equula* were CUA (Leu), AUC (Ile), CUC (Leu), GCC (Ala), ACC (Thr) and AUA (Met).

### ﻿Selection pressure analysis

**Figure 4.**
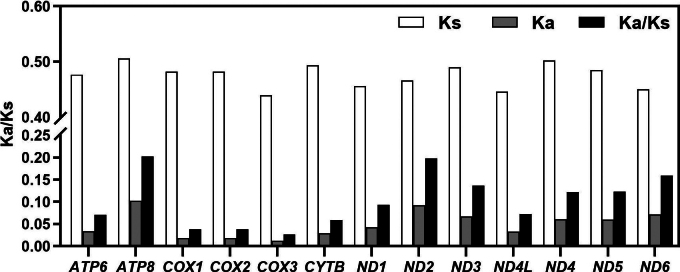
Mean Ka, Ks, and Ka/Ks values of 13 protein-coding genes from six Leiognathidae species.

The selection pressure on 13 PGCs of six Leiognathidae species was quantified by computing the ratio of non-synonymous substitutions to synonymous substitutions (Ka/Ks). The Ka/Ks ratios of all PCGs were significantly lower than one (Fig. 4), suggesting that all PCGs were subject to a strong purifying selection pressure in these species (Yang and Bielawski 2000). Among the PCGs, the ATP8 and COX3 genes showed the highest (0.2019) and lowest (0.0265) values of Ka/Ks, respectively.

### ﻿Transfer RNA and ribosomal RNA genes and control region

**Figure 5.**
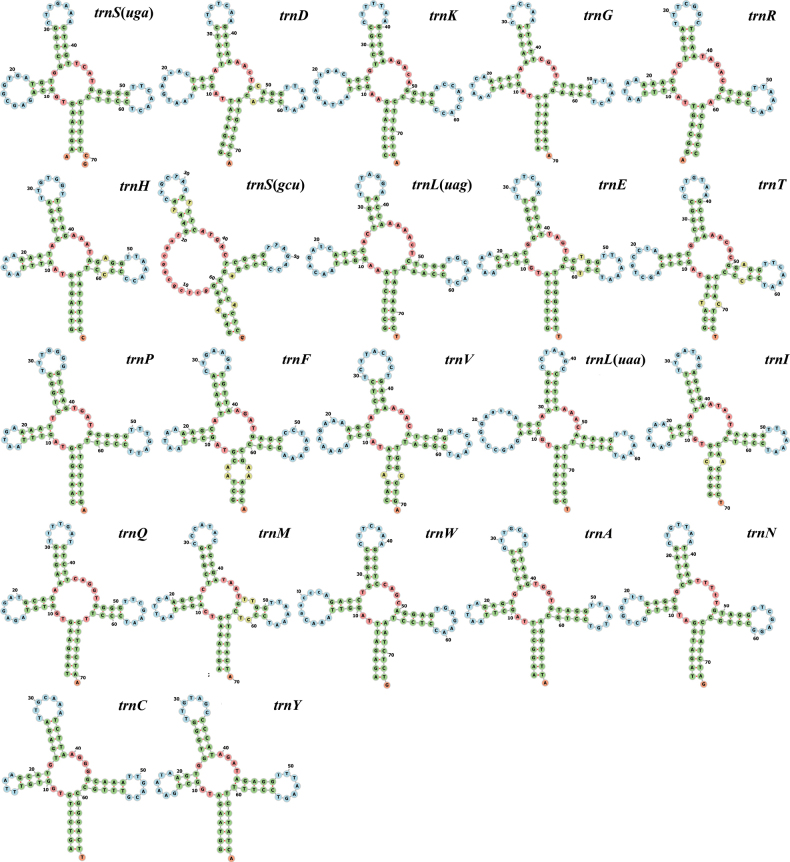
Secondary structure of the 22 tRNA genes in the mitochondrial genome of *L.equula*.

The mitogenome of *L.equula* contained 22 tRNA genes ranging from 67 to 75 bp in size, representing 9.5% (1552 bp) of the entire mitogenome (Suppl. material 1: table S7). Of the 22 tRNA genes, 14 were located on the H strand, and eight tRNA genes were located on the L strand (Suppl. material 1: table S8). All tRNA genes were predicted to fold into the typical cloverleaf secondary structure, except *trnS* (gcu), which lacked the dihydrouridine (DHU) arm (Fig. 5). The A+T content of the 22 tRNA genes was 57.60%, with a positive A+T skew (0.028) and G+C skew (0.065).

The two rRNA genes, *12S* and *16S* rRNA genes, were 949 bp and 1694 bp in length, respectively (Suppl. material 1: table S7). The rRNA genes were located between *trnF* (gaa) and *trnL* (uaa), separated from each other by *trnV* (uac) (Fig. 2, Suppl. material 1: table S7), as observed in other vertebrates. The A+T and G+C content of the two rRNA genes was 53.69% and 46.31%, respectively, with an A+T skew and a G+C skew of 0.29 and -0.10, respectively. These values indicate a clear bias in favor of the utilization of A and C nucleotides.

**Figure 6.**
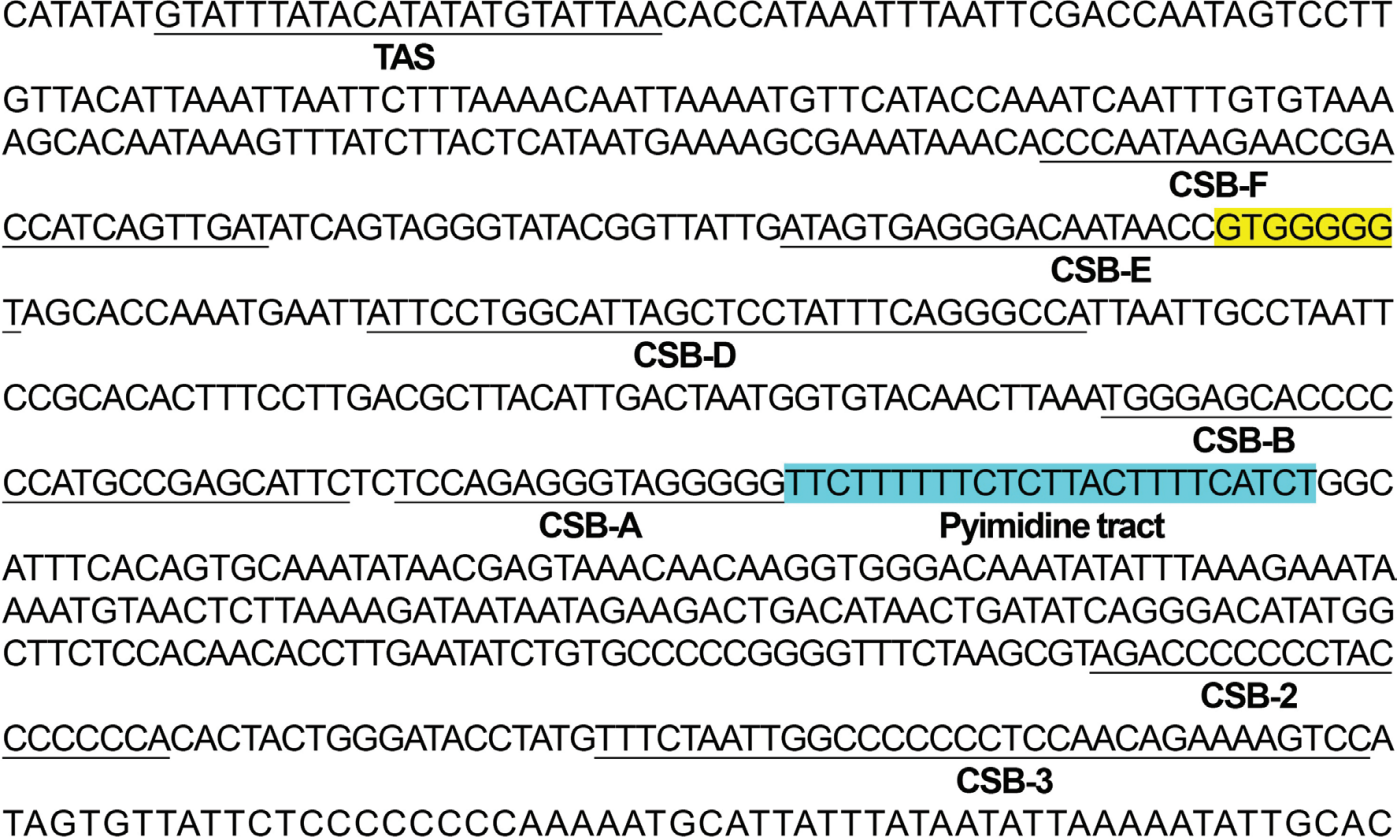
Structure and sequence of the control region of the *L.equula* mitochondrial genome. The termination-associated sequence (TAS), central conserved blocks (CSB-F, CSB-E, CSB-D, CSB-B, and CSB-A), and conserved sequence block domains (CSB-2 and CSB-3) are underlined.

The control region of *L.equula* is located between *trnP* (ugg) and *trnF* (gaa), with a total length of 727 bp (Fig. 2, Suppl. material 1: table S7). A termination-associated sequence (TAS), central conserved sequence block (CSB) domains containing five conserved sequence blocks (CSB-F, CSB-E, CSB-D, CSB-B, and CSB-A), and a variable domain consisting of two conserved sequence blocks (CSB-2 and CSB-3) were identified in the control region of the *L.equula* mitogenome through a homology search (Fig. 6). The G-box (GTGGGGG) was identified in the CSB-E, which exhibited the highest conservation across teleost fish. Additionally, a pyrimidine tract (TTCTTTTTTCTCTTACTTTTCATCT) was identified following the CSB-A, which was also present in other Leiognathidae species (Accession numbers from GenBank MG677547 and NC_057225).

### ﻿Phylogenetic analysis

**Figure 7.**
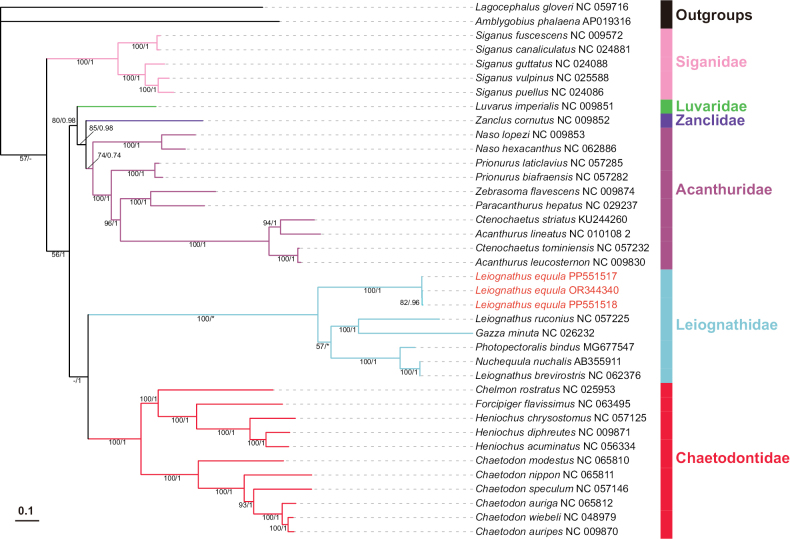
The position of Leiognathidae in the Bayesian inference (BI) and maximum likelihood (ML) phylogenetic tree based on the amino acid sequences of 13 protein-coding genes of the mitochondrial genome and their groupings, clades, ecotypes, and lengths. The numbers above or below branches indicate ML bootstrap values and Bayesian posterior probabilities, respectively; “*” indicates absence from the maximum clade credibility tree; “-” indicates no support value. *L.brevirostris* (NC 026232) should be a species of *Nuchequula*, and *L.ruconius* (NC 057225) is not classified under *Leiognathus* in the NCBI database.

The position of Leiognathidae in the molecular phylogenetic trees was reconstructed on the basis of 13 concatenated PCGs using the ML and BI methods. Phylogenetic analyses conducted using BI and ML yielded a consistent topology. The phylogenetic trees divided the Leiognathidae family into three distinct clades (Fig. 7). *Nuchequulanuchalis* (Temminck and Schlegel 1845) and *Leiognathusbrevirostris* (Valenciennes, in Cuvier and Valenciennes 1835) are grouped together with nearly equal branch lengths, indicating that the molecular record of *L.brevirostris* is likely also *Nuchequula*, supporting previous studies (Chakrabarty and Sparks 2007; Chakrabarty et al. 2010; Gao et al. 2020). *Nuchequula* spp. and *Photopectoralisbindus* (Valenciennes, in Cuvier and Valenciennes 1835) are sister branches clustered with single branches of *L.equula*. *Leiognathusruconius* (Hamilton, 1822) (now *Deveximentumruconius*) and *Gazzaminuta* (Bloch, 1795) forms another branch, they are the mandible vertical and presence of strongly caniniform teeth type of the family, respectively.

## ﻿Discussion

The morphometric and meristic values recorded in the present study were similar to those reported in previous studies on this species. But the number of pored scales in the lateral line (Table 1) was slightly higher than that previously reported. This difference is attributable to the high variability within the species, random pattern of scales, and the deciduous nature of ponyfish scales in preservation (Chakrabarty and Sparks 2007). Furthermore, accurate counting is challenging owing to the small size and degree of embedment of scales. Additionally, the preorbital depth expressed as percent head length (Table 1) is demonstrably higher, which may also be attributed to interspecific differences.

The genus *Leiognathus* currently includes *L.equula* and *L.robustus* (Sparks and Chakrabarty 2015) as well as several undescribed species (Chakrabarty and Sparks 2008; Chakrabarty et al. 2011). Four *Leiognathus* species are currently verified in Eschmeyer’s Catalog of Fishes (Fricke et al. 2024): *L.bindoides* (Bleeker, 1851), *L.equula* (Forsskål, in Niebuhr 1775), *L.parviceps* (Valenciennes, in Cuvier and Valenciennes 1835), and *L.robustus*Sparks and Dunlap 2004. However, *L.parviceps* is closely related to or conspecific with *Equulitesleuciscus* (Günther, 1860) (Sparks et al. 2005), and *L.bindoides* is considered a junior synonym of *L.bindus* (Weber and de Beaufort 1931) (=*Photopectoralisbindus*). But these species are not as large or strong as *L.equula*. Of the four *Leiognathus* species, *L.equula* and *L.robustus* are the better documented and more recently described species, respectively.

*Leiognathusequula*, the type species of *Leiognathus*, differs from *L.robustus*, which has been described in various reports (Sparks and Dunlap 2004; Chakrabarty and Sparks 2008, 2022), with respect to the presence of a pronounced hump (vs. absence of an occipital hump), strongly curved, creating the image of an arched back (vs. the presence of a mildly sloping predorsal proﬁle), slight bulge above orbit (vs. a distinct preorbital protuberance), and a nuchal spine that is exposed and projecting, particularly distally (vs. not exposed in lateral view). This species can be distinguished from other large leiognathid species such as *Aurigequulafasciata* and *Aurigequulastriatus* (James and Badrudeen, 1990) on the basis of its shorter second dorsal and anal fin spines, asquamate scale nuchal region, straighter dorsal head profile, and pigmentation pattern.

The *COX1* gene had a GTG start codon. Other Leiognathidae species have also been reported to use this non-standard start codon (Shi et al. 2018; Sui et al. 2019). Six PCGs exhibited an incomplete stop codon, a common feature of vertebrate PCGs that is thought to be completed by polyadenylation after transcription (Ojala et al. 1981). The 22 tRNA genes exhibited a distribution pattern similar to that observed in other Leiognathidae species (Shi et al. 2018; Sui et al. 2019). The secondary structure of *trnS* (gcu) lacked the dihydrouridine (DHU) arm. This finding is consistent with observations in all bony fish mitogenomes (Yang et al. 2018; Wang et al. 2022).

Previous phylogenetic studies on Leiognathidae did not use the mitochondrial genome. Studies on the mitochondrial genome of Leiognathidae species have been limited. The mitochondrial genome data of only five Leiognathidae species are available in the NCBI database (acquisition number: AB355911, MG677547, NC_026232, NC_057225, NC_062376; accessed February 6, 2024). There are two entries for *Leiognathus*: *L.ruconius* (acquisition number: NC_057225) and *L.brevirostris* (acquisition number: NC_062376). The former was identified as *Deveximentumruconius* (Sui et al. 2019; Fricke et al. 2024), whereas the latter is actually a species of *Nuchequula* (Chen and Fang 1999; Chakrabarty et al. 2010). The results of the phylogenetic analysis corroborate the aforementioned conclusions. In the present study, the species *L.ruconius* (NC 057225) did not form a clade with *L.equula* but instead formed a sister group with *G.minuta*. The species *L.brevirostris* (NC 026232), which is evidently not a *Leiognathus* species, clustered with *N.nuchalis*. Although the phylogenetic tree in the present study contained only six species, they belong to five different genera of the Leiognathidae. The phylogenetic characteristics above are consistent with previous findings (Sparks and Chakrabarty 2015).

Previous single-gene phylogenetic studies suggest that most genera within the family Leiognathidae are monophyletic (Ikejima et al. 2004; Sparks and Dunlap 2004; Sparks et al. 2005; Seah et al. 2008; Chakrabarty et al. 2011; Seth and Barik 2021). However, they are still nested within *Aurigequula* and *Leiognathus* (Sparks and Chakrabarty 2015). The phylogenetic analysis based on mitochondrial genome sequences (Fig. 7) and single gene sequences (*16S* RNA and *COX1*, Suppl. material 1: figs S3, S4) showed that the family Leiognathidae is monophyletic, whereas the phylogenetic analysis based on the single gene *ND5* (Suppl. material 1: fig. S5) showed the opposite. This can be attributed to the fact that the sequences of the three unidentified species of *Leiognathus* are not grouped with those of the other known species of Leiognathidae.

In the present study, the phylogenies based on mitochondrial genome sequences showed that Leiognathidae are most closely related to Chaetodontidae, forming a sister group. This finding is consistent with the osteological evidence (Gill and Leis 2019; Gill and Michalski 2020). Moreover, the families Acanthuridae, Luvaridae, and Zanclidae formed a separate clade, which forms a sister group to the Leiognathidae and Chaetodontidae clades. This finding is corroborated by previous findings on the time-calibrated phylogeny of the bony fish species (Betancur-R et al. 2017). Whole mitochondrial genome phylogeny suggested with high support that Leiognathidae and Chaetodontidae comprise the order Chaetodontiformes, whereas Acanthuridae, Luvaridae and Zanclidae comprise the order Acanthuriformes (Fig. 7).

## ﻿Conclusions

In the present study, samples of *L.equula* were collected from different regions of China. The species were identiﬁed using both morphological and molecular characteristics. Phylogenies based on the amino acid sequences of 13 protein-coding genes and two single gene sequences (*16S* RNA and *COX1*) but not that based on the single gene *ND5*, indicated that Leiognathidae is a monophyletic family. The phylogenetic trees show that the family Leiognathidae is divided into three clades. Notably, the family Leiognathidae formed was placed as a sister group to the family Chaetodontidae. In the present study, the mitochondrial genome sequence of *L.equula* in the family Leiognathidae was obtained using shallow genome skimming. *L.equula* occupied a basal branch of the Leiognathidae phylogenetic tree; thus, the study provides essential data for the study of the complete mitochondrial genome phylogeny of Leiognathidae.

## ﻿Additional information

### Author ORCIDs

Jiajie Chen https://orcid.org/0000-0001-7487-231X

Xiaodong Wang https://orcid.org/0000-0001-9424-7896

Sheng Zeng https://orcid.org/0009-0001-0943-3772

Wei Tian https://orcid.org/0009-0008-5272-0700

Deyuan Yang https://orcid.org/0000-0003-3735-9909

Jinqing Ye https://orcid.org/0009-0009-6503-1477

Junsheng Zhong https://orcid.org/0000-0002-9145-7473

Chaopeng Jiang https://orcid.org/0009-0004-0957-231X

### Data availability

All of the data that support the findings of this study are available in the main text or Supplementary Information.

## Supplementary materials

Supplementary material 1 Supplementary file Data type zip Explanation note **figure S1.***Leiognathusequula* was on sale at the landing ports (above) and fish markets (below) in Qinglan, Hainan, on November 9, 2023. **figure S2.** Dorsal head slightly triangular, *L.equula* DHS14327. Arrow indicates the nuchal spine. **figure S3.** Phylogenetic trees of Leiognathidae based on the *16S rRNA* dataset constructed using the Bayesian inference and maximum likelihood methods. **figure S4.** Phylogenetic trees of Leiognathidae based on the *COX1* dataset constructed using the Bayesian inference and maximum likelihood methods. **figure S5.** Phylogenetic trees of Leiognathidae based on the *ND5* dataset constructed using the Bayesian inference and maximum likelihood methods. **table S1.** Collection data for eight specimens of *L.equula*. **table S2.** Taxa used in the *16S rRNA* and *COX1* molecular phylogenetic analysis. **table S3.** Taxa sampled for the *ND5* phylogenetic analysis. **table S4.** Information on raw data and cleaned data from three specimens. **table S5.** List of mitogenomes included in the present study and their base composition and GenBank accession numbers. **table S6.** Morphometric and meristic data of *L.equula* in this study. **table S7.** Information on each gene fragment of *L.equula*. **table S8.** Base composition of the *L.equula* mitochondrial genome. **table S9.** Codon number and relative synonymous codon usage of *L.equula* mitochondrial protein-coding genes. https://binary.pensoft.net/file/1189578This dataset is made available under the Open Database License (http://opendatacommons.org/licenses/odbl/1.0/). The Open Database License (ODbL) is a license agreement intended to allow users to freely share, modify, and use this Dataset while maintaining this same freedom for others, provided that the original source and author(s) are credited.Jiajie Chen, Xiaodong Wang, Sheng Zeng, Wei Tian, Deyuan Yang, Jinqing Ye, Junsheng Zhong, Chaopeng Jiang
