# Supplementary material for: ﻿Morphometric and phylogenetic analysis of a commercial fish Leiognathusequula (Teleostei, Leiognathidae)
Source: Zookeys. 2024 Dec 4;1219:249–70. doi: 10.3897/zookeys.1219.130546 (PMC11635357; doi:10.3897/zookeys.1219.130546)
Supplement: Supplementary material 1 — Supplementary file [file zookeys-1219-249_article-130546__-s001.zip › 130546_0R-2-A_Table_S1-S9-Complete_L._equula.pdf]

Table S1. Collection data for eight specimens of *L. equula*.

Voucher number	Date	Collection locality	Accession No.
DHS14325	Dec. 5, 2021	Changjiang, Hainan Island, China	——
DHS14326			——
DHS14327			OR344340
DHS17273	Apr. 30, 2022		——
DHS14500	Oct. 21, 2021	Red Bay, Guangdong, China	——
DHS19056	Oct. 26, 2023	Yangxi, Guangdong, China	PP551517
DHS22489	Nov. 7, 2023	Hongsha Fish Market, Hainan Island, China	——
DHS22490			PP551518

Table S2. Taxa used in *16S* and *COX1* molecular phylogenetic analysis, including collection locality, tissue voucher number, and GenBank accession numbers.

Species	Collection locality	Voucher number	*16S*	*COX1*	Reference
**Carangidae**					
Carangoides equula	Japan	KE-1	AY541670	AY541645	Sparks and Dunlap 2004, Seah et al. 2008
Carangoides malabaricus	Philippines	CM-1	AY541671	AY541646	Sparks and Dunlap 2004
Selar crumenophthalmus	Japan	SC-1	AY541672	AY541647	Sparks and Dunlap 2004
**Gerridae**					
Gerres abbreviatus	Philippines	GAB-1	AY541667	AY541642	Sparks and Dunlap 2004, Seah et al. 2008
Gerres equulus	Japan	GE-1	AY541668	AY541643	Sparks and Dunlap 2004, Seah et al. 2008
Gerres filamentosus	Philippines	CM-1	AY541669	AY541644	Sparks and Dunlap 2004
**Leiognathidae**					
Aurigequula fasciata	Philippines	LF-2P	AY541655	AY541630	Sparks and Dunlap 2004, Seah et al. 2008, Chakrabarty et al. 2011
Aurigequula fasciata	Madagascar		DQ027952	DQ028021	Chakrabarty et al. 2011
Aurigequula longispina	Singapore		DQ027944	DQ028014	Chakrabarty et al. 2011
Aurigequula longispina	Tinggi Island, Malaysia	MSUKM0004	EU366331		Seah et al. 2008
Aurigequula sp.	Madagascar		DQ027942	DQ028012	Chakrabarty et al. 2011
Aurigequula striatus	Sri Lanka		DQ027945	DQ028015	Chakrabarty et al. 2011
Deveximentum cf. insidiator	Sri Lanka		DQ027971	DQ028041	Sparks et al. 2005, Seah et al. 2008, Chakrabarty et al. 2011
Deveximentum cf. ruconius	Sri Lanka		DQ027973	DQ028043	Chakrabarty et al. 2011
Deveximentum hanedai	Tinggi Island, Malaysia	MSUKM0005	EU366332		Seah et al. 2008
Deveximentum indicium	Philippines	SI-1P	AY541665	AY541640	Sparks and Dunlap 2004, Seah et al. 2008
Deveximentum indicium	Sri Lanka		DQ027970		Sparks et al. 2005, Seah et al. 2008
Deveximentum indicium	Perhentian Island, Malaysia	MSUKM0009	EU366336		Seah et al. 2008
Deveximentum insidiator	Taiwan, China		HQ993133	HQ993161	Chakrabarty et al. 2011
Deveximentum insidiator	India	EBRC/ZSI/F-10957	MK625082	MK610265	Seth and Barik 2021
Deveximentum interruptum	India	EBRC/ZSI/F-10958	MK625068	MK619231	Seth and Barik 2021
Deveximentum megalolepis	Philippines	SM-1P	AY541666	AY541641	Sparks and Dunlap 2004, Seah et al. 2008, Chakrabarty et al. 2011
Deveximentum megalolepis	Australia		DQ027972		Sparks et al. 2005, Seah et al. 2008
Deveximentum megalolepis	Tigbauan, Philippines	D-0008	DQ648432		Dunlap et al. 2007, Seah et al. 2008
Deveximentum megalolepis	Tinggi Island, Malaysia	MSUKM0010	EU366337		Seah et al. 2008
Deveximentum sp.	Madagascar		DQ027967	DQ028037	Chakrabarty et al. 2011

Species	Collection locality	Voucher number	*16S*	*COX1*	Reference
Equulites elongatus	Japan	LE-1J	AY541652	AY541627	Sparks and Dunlap 2004, Chakrabarty et al. 2011
Equulites laterofenestra	Perhentian Island, Malaysia	MSUKM0015	EU366342		Seah et al. 2008, Chakrabarty et al. 2011
Equulites leuciscus	Philippines	LL-1P	AY541657	AY541632	Sparks and Dunlap 2004, Seah et al. 2008, Chakrabarty et al. 2011
Equulites leuciscus	Madagascar		DQ027964	DQ028034	Sparks et al. 2005, Seah et al. 2008, Chakrabarty et al. 2011
Equulites leuciscus	Japan		DQ027965		Sparks et al. 2005, Seah et al. 2008
Equulites leuciscus	Perhentian Island, Malaysia	MSUKM0012	EU366339		Seah et al. 2008
Equulites leuciscus	Tinggi Island, Malaysia	MSUKM0013	EU366340		Seah et al. 2008
Equulites leuciscus	Tinggi Island, Malaysia	MSUKM0021	EU366348		Seah et al. 2008
Equulites leuciscus	India	EBRC/ZSI/F-10960	MK640616	MK689369	Seth and Barik 2021
Equulites lineolatus	Sri Lanka		DQ027966	DQ028036	Chakrabarty et al. 2011
Equulites lineolatus	Malaysia		HQ993124	HQ993152	Chakrabarty et al. 2011
Equulites lineolatus	India	EBRC/ZSI/F-10959	MK650845	MK672879	Seth and Barik 2021
Equulites rivulatus	Japan	LR-1J	AY541661	AY541636	Sparks and Dunlap 2004, Seah et al. 2008, Chakrabarty et al. 2011
Equulites sp.	Madagascar		DQ027963	DQ028033	Chakrabarty et al. 2011
Equulites stercorarius	Thailand		HQ993126	HQ993153	Chakrabarty et al. 2011
Equulites stercorarius	Philippines	LST-1P	AY541663	AY541638	Sparks and Dunlap 2004, Seah et al. 2008
Equulites stercorarius	Perhentian Island, Malaysia	MSUKM0007	EU366334		Seah et al. 2008
Equulites stercorarius	Tinggi Island, Malaysia	MSUKM0018	EU366345		Seah et al. 2008
Eubleekeria jonesi	Philippines	LJ-1P	AY541656	AY541631	Sparks and Dunlap 2004, Seah et al. 2008
Eubleekeria jonesi	Thailand		HQ993116	HQ993144	Chakrabarty et al. 2011
Eubleekeria jonesi	Tinggi Island, Malaysia	MSUKM0008	EU366335		Seah et al. 2008
Eubleekeria jonesi	Perhentian Island, Malaysia	MSUKM0019	EU366346		Seah et al. 2008
Eubleekeria splendens	Philippines	LP-1P	AY541660	AY541635	Sparks and Dunlap 2004
Eubleekeria splendens	Taiwan, China		HQ993117	HQ993145	Chakrabarty et al. 2011
Eubleekeria splendens	Philippines	LS-2P	AY541662	AY541637	Sparks and Dunlap 2004, Seah et al. 2008
Eubleekeria splendens	India	EBRC/ZSI/F-10963	MK621021	MK610267	Seth and Barik 2021
Gazza achlamys	Philippines	GA-1	AY541648	AY541623	Sparks and Dunlap 2004, Chakrabarty et al. 2011
Gazza cf. rhombea	Taiwan, China		HQ993114	HQ993142	Chakrabarty et al. 2011
Gazza minuta	Philippines	GM-1	AY541650	AY541624	Sparks and Dunlap 2004, Seah et al. 2008, Chakrabarty et al. 2011
Gazza minuta	Sri Lanka		DQ027936		Sparks et al. 2005, Seah et al. 2008

Species	Collection locality	Voucher number	*16S*	*COX1*	Reference
Gazza minuta	Sri Lanka		DQ027937		Sparks et al. 2005, Seah et al. 2008
Gazza minuta	Tigbauan, Philippines	D-0030	DQ648428		Dunlap et al. 2007, Seah et al. 2008
Gazza minuta	Perhentian Island, Malaysia		EU366338		Seah et al. 2008
Gazza minuta	Tinggi Island, Malaysia		EU366347		Seah et al. 2008
Gazza minuta	India	EBRC/ZSI/F-10961	MK644024	MK630704	Seth and Barik 2021
Gazza squamiventralis	Madagascar		DQ027938	DQ028008	Chakrabarty et al. 2011
Karalla daura	Sri Lanka		DQ027955	DQ028026	Sparks et al. 2005, Seah et al. 2008.Chakrabarty et al. 2011
Karalla daura	Sri Lanka		DQ027954		Sparks et al. 2005, Seah et al. 2008
Karalla daura	Tinggi Island, Malaysia		EU366333		Seah et al. 2008
Karalla daura	India	EBRC/ZSI/F-10964	MK644026	MK689370	Seth and Barik 2021
Karalla dussumieri	Sri Lanka		DQ027959	DQ028030	Chakrabarty et al. 2011
Karalla dussumieri	India	EBRC/ZSI/F-10965	MK636537	MK630703	Seth and Barik 2021
Leiognathus bindus	Philippines	LB-1P	AY541652	AY541626	Sparks and Dunlap 2004, Seah et al. 2008, Chakrabarty et al. 2011
Leiognathus cf. *striatus*	Singapore		HQ993118	HQ993146	Chakrabarty et al. 2011
Leiognathus equula	Philippines	LEQ-1P	AY541653	AY541628	Sparks and Dunlap 2004, Seah et al. 2008
Leiognathus equula	Singapore	LEQ-2S	AY541654	AY541630	Sparks and Dunlap 2004, Seah et al. 2008
Leiognathus equula	Taiwan, China		DQ027948	DQ028018	Chakrabarty et al. 2011, Seah et al. 2008
Leiognathus equula	Japan		DQ027947		Sparks et al. 2005, Seah et al. 2008
Leiognathus equula	Perhentian Island, Malaysia	MSUKM0014	EU366341		Seah et al. 2008
Leiognathus equula	India	EBRC/ZSI/F-10966	MK644023	MK689371	Seth and Barik 2021
Leiognathus equula	Hainan Island, China	DHS14327	OR344340	OR344340	This study
Leiognathus equula	Yangxi, Guangdong, China	DHS19056	PP552627	PP552627	This study
Leiognathus equula	Hainan Island, China	DHS22500	PP552628	PP552628	This study
Leiognathus robustus	Singapore	LEQ-1S	AY541664	AY541639	Sparks and Dunlap 2004
Leiognathus robustus	Singapore		DQ027953	DQ028023	Chakrabarty et al. 2011
Leiognathus sp.	Fiji		DQ027940	DQ028010	Chakrabarty et al. 2011
Nuchequula blochii	Kuala Kedah, Malaysia	MSUKM0130	EU741826		Chakrabarty et al. 2011
Nuchequula cf. gerreoides	Sri Lanka		DQ027957	DQ028027	Sparks et al. 2005, Seah et al. 2008, Chakrabarty et al. 2011
Nuchequula decora	Australia		DQ027956	DQ028026	Sparks et al. 2005, Seah et al. 2008, Chakrabarty et al. 2011

Species	Collection locality	Voucher number	*16S*	*COX1*	Reference
Nuchequula decora	Perhentian Island, Malaysia	MSUKM0001	EU366328		Seah et al. 2008
Nuchequula decora	Tinggi Island, Malaysia	MSUKM0016	EU366343		Seah et al. 2008
Nuchequula gerreoides	India	EBRC/ZSI/F-10956	MK621022	MK610266	Seth and Barik 2021
Nuchequula longicornus	Thailand		HQ993121	HQ993150	Chakrabarty et al. 2011
Nuchequula mannusella	Taiwan, China		HQ993119	HQ993147	Chakrabarty et al. 2011
Nuchequula mannusella	Tinggi Island, Malaysia	MSUKM0003	EU366330		Seah et al. 2008
Nuchequula nuchalis	Japan	LN-1J	AY541658	AY541633	Sparks and Dunlap 2004, Chakrabarty et al. 2011
Photopectoralis aureus	Philippines	LA-1P	AY541651	AY541626	Sparks and Dunlap 2004, Chakrabarty et al. 2011
Photopectoralis bindus	Perhentian Island, Malaysia	MSUKM0002	EU366330		Seah et al. 2008
Photopectoralis bindus	Tinggi Island, Malaysia	MSUKM0017	EU366344		Seah et al. 2008
Photopectoralis bindus	India	EBRC/ZSI/F-10967	MK636538	MK619232	Seth and Barik 2021
Photopectoralis cf. *aureus*	Thailand		HQ993128	HQ993156	Chakrabarty et al. 2011
Photopectoralis sp.	Taiwan, China		DQ027962	DQ028032	Sparks et al. 2005, Seah et al. 2008, Chakrabarty et al. 2011
Photopectoralis panayensis	Philippines	LH-1P	AY541659	AY541634	Sparks and Dunlap 2004, Chakrabarty et al. 2011
Photopectoralis sp.	Taiwan, China		DQ027961		Sparks et al. 2005, Seah et al. 2008

Reference:

Chakrabarty P, Davis MP, Smith WL, Baldwin ZH, Sparks JS (2011) Is sexual selection driving diversification of the bioluminescent ponyfishes (Teleostei: Leiognathidae)? Molecular Ecology 20(13): 2818-2834. https://doi.org/10.1111/j.1365-294x.2011.05112.x

Dunlap PV, Ast, JC, Kimura S, Fukui A, Yoshino T, Endo H (2007) Phylogenetic analysis of host-symbiont specificity and codivergence in bioluminescent symbioses. Cladistics 23(5): 507-532. https://doi.org/10.1111/j.1096-0031.2007.00157.x

Seah YG, Ghaffar MA, Usup G (2008) Phylogeny of Ponyfishes from Coastal Waters of the South China Sea. Journal of Applied Biological Sciences 2(3): 125-132. https://dergipark.org.tr/en/pub/jabs/issue/34967/387982

Seth JK, Barik TK (2021) DNA barcoding of the family: Leiognathidae in the water of Bay of Bengal, Odisha coast, India based on 16s rRNA and COI gene sequences. Thalassas: An International Journal of Marine Sciences 37: 831-840. https://doi.org/10.1007/s41208-021-00324-1

Sparks JS, Dunlap PV (2004) A clade of non-sexually dimorphic ponyfishes (Teleostei: Perciformes: Leiognathidae): phylogeny, taxonomy, and description of a new species. American Museum Novitates 3459: 1–21. https://doi.org/10.1206/0003-0082(2004)459%3c0001:ACONDP%3e2.0.CO;2

Sparks JS, Dunlap PV, Smith WL (2005) Evolution and diversification of a sexually dimorphic luminescent system in ponyfishes (Teleostei: Leiognathidae), including diagnoses for two new genera. Cladistics, 21(4), 305-327. https://doi.org/10.1111/j.1096-0031.2005.00067.x

Table S3. Taxa used in *16S* Taxa sampled for the *ND5* phylogenetic analysis with corresponding GenBank accession numbers. Collection localities are provided for ponyfish taxa. Asterisks denote taxa that include *ND4* and tRNAs with *ND5* fragment.

Species	Collection locality	Voucher specimens	Accession No.	Reference
**Carangidae**				
Carangoides malabaricus	Philippines		DQ028051	Sparks et al. 2005
Scomberoides lysan			DQ028052	Sparks et al. 2005
Trachinotus ovatus			DQ028050	Sparks et al. 2005
**Gerridae**				
Gerres equulus	Japan		DQ028053	Sparks et al. 2005, Chakrabarty et al. 2011
**Leiognathidae**				
Aurigequula sp.	Madagascar	AMNH 120338	DQ028069	Sparks et al. 2005, Chakrabarty et al. 2011
Deveximentum hanedai	Trang, Thailand	MRIT-P70	AB100022	Sparks et al. 2005, Chakrabarty et al. 2011
Deveximentum indicium	Panay Island, Philippines	NSMT-P62519	AB100023	Ikejima et al. 2004, Sparks et al. 2005, Chakrabarty et al. 2011
Deveximentum megalolepis	Trang, Thailand	MRIT-P153	AB100024	Ikejima et al. 2004, Chakrabarty et al. 2011
Deveximentum megalolepis	Australia	WI-02-11	DQ028076	Ikejima et al. 2004, Sparks et al. 2005, Chakrabarty et al. 2011
Equulites elongatus	Kanagawa, Japan	NSMT-P62514	AB100016	Sparks et al. 2005
Equulites rivulatus	Kanagawa, Japan	NSMT-P62517	AB100019	Ikejima et al. 2004, Sparks et al. 2005, Chakrabarty et al. 2011
Equulites stercorarius	Trang, Thailand	MRIT- P152	AB100021	Ikejima et al. 2004, Sparks et al. 2005, Chakrabarty et al. 2011
Eubleekeria splendens	Panay Island, Philippines	NSMT-P62518	AB100020	Ikejima et al. 2004, Sparks et al. 2005, Chakrabarty et al. 2011
Gazza achlamys	Panay Island, Philippines	NSMT-P62512	AB100025	Ikejima et al. 2004, Sparks et al. 2005, Chakrabarty et al. 2011
Gazza dentex	Trang, Thailand	MRIT-P154	AB100027	Ikejima et al. 2004, Chakrabarty et al. 2011
Gazza minuta	Panay Island, Philippines	NSMT-P62513	AB100027	Ikejima et al. 2004, Sparks et al. 2005, Chakrabarty et al. 2011
Gazza minuta	Sri Lanka	LEI 6 SL	DQ028066	Ikejima et al. 2004, Chakrabarty et al. 2011
Gazza sp.	Madagascar	AMNH 120340	DQ028062	Sparks et al. 2005
Gazza sp.	Madagascar	WLSMadT240	DQ028063	Sparks et al. 2005
Gazza sp.	Madagascar	AMNH 120364	DQ028064	Sparks et al. 2005
Gazza sp.	Madagascar	AMNH 119978	DQ028065	Sparks et al. 2005
Karalla dussumieri	Sri Lanka	LEI 10 SL	DQ028073	Sparks et al. 2005
Karalla dussumieri	Sri Lanka	AMNH 234763	DQ028074	Sparks et al. 2005
Karalla dussumieri	Sri Lanka	LEI 18 SL	DQ028075	Sparks et al. 2005, Chakrabarty et al. 2011

Species	Collection locality	Voucher specimens	Accession No.	Reference
Leiognathus equula	Panay Island, Philippines	NSMT-P62515	AB100017	Sparks et al. 2005
Leiognathus equula	Hainan Island, China	DHS14327	OR344340	This study
Leiognathus equula	Yangxi, Guangdong, China	DHS19056	PP551517	This study
Leiognathus equula	Hainan Island, China	DHS22490	PP551518	This study
Leiognathus sp.	Fiji	KU 4440	DQ028067	Sparks et al. 2005, Chakrabarty et al. 2011
Leiognathus sp.	Madagascar	AMNH 119979	DQ028068	Sparks et al. 2005
Leiognathus sp.	Madagascar	AMNH 120356	DQ028070	Sparks et al. 2005
Nuchequula cf. flavaxilla	Philippines	AMNH 122171	DQ028071	Sparks et al. 2005, Chakrabarty et al. 2011
Nuchequula cf. gerreoides	Sri Lanka	AMNH 234765	DQ028072	Sparks et al. 2005, Chakrabarty et al. 2011
Nuchequula decora	Trang, Thailand	MRIT-P151	AB100015	Ikejima et al. 2004, Sparks et al. 2005, Chakrabarty et al. 2011
Nuchequula nuchalis	Chiba, Japan	NSMT-P62516	AB100028	Ikejima et al. 2004, Sparks et al. 2005, Chakrabarty et al. 2011
Nuchequula pan	Trang, Thailand	MRIT-P67	AB100018	Ikejima et al. 2004, Sparks et al. 2005, Chakrabarty et al. 2011

Reference

Chakrabarty P, Davis MP, Smith WL, Baldwin ZH, Sparks JS (2011) Is sexual selection driving diversification of the bioluminescent ponyfishes (Teleostei: Leiognathidae)? Molecular Ecology 20(13): 2818-2834. https://doi.org/10.1111/j.1365-294x.2011.05112.x

Ikejima K, Ishiguro NB, Wada M, Kita-Tsukamoto K, Nishida M (2004) Molecular phylogeny and possible scenario of ponyfish (Perciformes: Leiognathidae) evolution. Molecular Phylogenetics and Evolution 31(3): 904-909. https://doi.org/10.1016/j.ympev.2003.10.006

Sparks JS, Dunlap PV, Smith WL (2005) Evolution and diversification of a sexually dimorphic luminescent system in ponyfishes (Teleostei: Leiognathidae), including diagnoses for two new genera. Cladistics, 21(4), 305-327. https://doi.org/10.1111/j.1096-0031.2005.00067.x

Table S4. The information of raw data and cleaned data from 3 specimen.

Voucher numbers	Raw data			Cleaned data		
	Total bases (Gbp)	Total reads	Duplicate reads (%)	Total bases (Gbp)	Total reads	Duplicate reads (%)
DHS14327_1	5.3	35777359	14.9	5.3	35370424	14.3
DHS14327_2			15			14.6
DHS19056_1	2.6	17632089	15.1	2.1	17190343	14.8
DHS19056_2			15.3			15.3
DHS22490_1	2.3	15710969	14.7	2.3	15535689	14.7
DHS22490_2			14.8			14.8

Table S5. List of mitogenomes included in the present study and their base composition and GenBank accession numbers.

NO.	Species	Size (bp)	A%	T%	G%	C%	A+T Content (%)	A+T Skew	G+C Skew	Accession No.
1	*Lagocephalus gloveri*	16446	27.6	25.1	16.5	30.8	52.7	0.048	-0.302	NC_059716
2	*Amblygobius phalaena*	16622	26.2	25.0	18.7	30.0	51.2	0.024	-0.231	AP019316
3	*Siganus fuscescens*	16491	28.1	25.4	16.8	29.8	53.5	0.050	-0.280	NC_009572
4	*Siganus canaliculatus*	16492	28.1	25.4	16.7	29.7	53.5	0.051	-0.281	NC_024881
5	*Siganus guttatus*	16505	29.3	25.7	15.7	29.3	55.0	0.064	-0.302	NC_024088
6	*Siganus vulpinus*	16505	28.9	25.5	16.0	29.6	54.4	0.064	-0.300	NC_025588
7	*Siganus puellus*	16504	28.6	25.6	16.3	29.5	54.2	0.056	-0.288	NC_024086
8	*Zanclus cornutus*	16521	26.4	26.3	17.6	29.7	52.7	0.002	-0.257	NC_009852
9	*Luvarus imperialis*	16497	26.8	25.5	17.6	30.1	52.3	0.025	-0.261	NC_009851
10	*Naso lopezi*	16542	28.9	26.2	15.9	29.0	55.1	0.048	-0.290	NC_009853
11	*Naso hexacanthus*	16611	29.0	25.8	16.1	29.1	54.8	0.059	-0.287	NC_062886
12	*Prionurus laticlavius*	16531	29.4	26.3	15.4	28.8	55.7	0.057	-0.302	NC_057285
13	*Prionurus biafraensis*	16552	29.2	26.0	15.6	28.8	55.2	0.058	-0.298	NC_057282
14	*Zebrasoma flavescens*	16503	28.9	25.5	16.1	29.5	54.4	0.062	-0.295	NC_009874
15	*Paracanthurus hepatus*	16498	28.6	26.4	16.3	28.6	55.0	0.039	-0.274	NC_029237
16	*Acanthurus leucosternon*	16434	29.3	26.4	15.8	28.5	55.7	0.052	-0.287	NC_009830
17	*Ctenochaetus tominiensis*	16442	29.2	26.3	15.8	28.6	55.5	0.052	-0.287	NC_057232
18	*Ctenochaetus striatus*	17272	29.4	26.5	15.8	28.3	55.9	0.051	-0.285	KU244260
19	*Acanthurus lineatus*	16532	29.8	26.6	15.5	28.1	56.4	0.058	-0.289	NC_010108
20	*Leiognathus ruconius*	16465	31.6	25.2	14.5	28.7	56.8	0.112	-0.327	NC_057225
21	*Gazza minuta*	16475	29.5	25.3	15.8	29.5	54.8	0.076	-0.303	NC_026232
22	*Leiognathus equula*	16398	30.7	24.2	15.0	30.1	54.9	0.117	-0.334	OR344340
23	*Leiognathus equula*	16399	30.6	24.3	15.0	30.1	54.9	0.117	-0.333	PP551517
24	*Leiognathus equula*	16395	30.7	24.2	15.0	30.1	54.9	0.117	-0.333	PP551518
25	*Photopectoralis bindus*	16517	29.9	25	15.1	30.1	54.9	0.089	-0.333	MG677547
26	*Nuchequula nuchalis*	15965	29.8	25.3	15.2	29.7	55.1	0.080	-0.322	AB355911
27	*Leiognathus brevirostris*	16465	29.6	25.5	15.3	29.6	55.1	0.074	-0.318	NC_062376
28	*Chelmon rostratus*	16538	28.9	28.1	16.2	26.8	57.0	0.015	-0.246	NC_025953
29	*Forcipiger flavissimus*	16600	27.8	26.9	16.9	28.4	54.7	0.017	-0.254	NC_063495
30	*Heniochus chrysostomus*	16650	28.6	25.5	16.2	29.6	54.1	0.059	-0.292	NC_057125
31	*Heniochus diphreutes*	16772	29.2	25.8	15.6	29.4	55.0	0.061	-0.305	NC_009871
32	*Heniochus acuminatus*	16584	29.0	26.0	15.8	29.2	55.0	0.054	-0.299	NC_056334
33	*Chaetodon modestus*	16490	28.0	28.7	16.7	26.5	56.7	-0.012	-0.227	NC_065810
34	*Chaetodon nippon*	16507	27.5	27.9	16.8	27.8	55.4	-0.006	-0.248	NC_065811
35	*Chaetodon speculum*	16513	27.8	26.6	16.4	29.1	54.4	0.022	-0.278	NC_057146
36	*Chaetodon auriga*	16527	28.2	26.3	16.3	29.2	54.5	0.034	-0.284	NC_065812
37	*Chaetodon wiebeli*	16523	27.9	26.4	16.6	29.1	54.3	0.027	-0.274	NC_048979
38	*Chaetodon auripes*	16530	27.9	26.4	16.6	29.2	54.3	0.027	-0.276	NC_009870

Table S6. Morphometric and meristic data of *L. equula* in this study.

**Counts and measurements**	DHS14325	DHS14326	DHS14327	DHS14500	DHS17273	DHS19056	DHS22489	DHS22490
**Standard length (mm)**	118.71	122.58	144.02	81.33	98.80	107.58	86.48	88.38
**Counts**								
Dorsal fin rays	VIII, 16	VIII, 16	VIII, 16	VIII, 16	VIII, 16	VIII, 16	VIII, 16	VIII, 16
Anal fin rays	III, 14	III, 14	III, 14	III, 14	III, 14	III, 14	III, 14	III, 14
Pectoral fin rays	20	20	20	20	20	20	20	20
Pored scales in lateral line	73	68	70	63	64	67	65	65
Vertebrae (precaudal+caudal)	9+14=23	9+14=23	9+14=23	9+14=23	9+14=23	9+14=23	9+14=23	9+14=23
**Measurements**								
**As % of standard length**								
Head length	34.09	31.47	32.79	33.27	31.62	35.91	33.92	32.59
Body depth (origin AF) (A)	55.35	54.66	52.95	59.56	53.60	57.77	56.30	54.72
Body depth (origin DF) (B)	56.45	57.48	55.40	58.27	54.76	60.38	56.61	57.33
Head width (max.)	12.77	13.22	11.87	11.82	11.52	13.01	11.88	12.22
Caudal peduncle length	5.07	5.64	5.01	6.73	5.81	5.74	5.52	4.66
Caudal peduncle depth	8.25	7.67	7.08	7.17	7.21	7.87	7.78	7.54
Caudal peduncle width	4.61	4.58	3.83	4.66	3.42	5.10	3.98	3.91
Pectoral-fin length	27.18	26.35	24.47	23.20	25.17	26.98	26.47	26.02
Pelvic-fin length	15.58	15.66	14.98	16.64	15.66	16.78	17.24	12.58
Dorsal fin base length	57.50	57.15	57.03	59.70	56.34	59.09	58.53	57.03
Anal fin base length	45.82	45.66	45.05	50.79	45.37	48.60	48.16	47.22
Predorsal length	52.83	52.86	51.73	52.27	50.22	54.32	52.39	53.29
Prepelvic length	43.58	40.23	43.57	39.78	38.05	44.85	37.62	40.69
Preanal length	58.57	56.40	59.36	54.79	54.71	58.90	57.03	57.49
**As % of head length**								
Snout length	36.35	40.37	34.77	33.26	35.31	36.40	34.74	39.76
Head width (max.)	37.46	42.00	36.21	35.51	36.43	36.24	35.02	37.50
Upper jaw length	16.41	18.33	15.65	15.08	17.16	15.79	16.57	16.32
Lower jaw length	18.26	23.10	18.53	19.33	21.19	14.99	15.10	16.04
Interorbital width	33.61	38.48	33.83	25.31	31.91	31.53	30.41	31.11
Orbit diameter	31.53	33.42	28.58	30.71	29.96	30.83	30.34	33.54
Preorbital depth	67.95	72.65	73.55	68.07	72.15	76.47	67.13	77.29

Table S7. Information on each gene fragment of *L. equula*

Name	Start	Stop	Strand	Length	Intergenic Region *	Codon
COX1	1	1551	H	1551	2	GTG/TAG
trnS(uga)	1554	1624	L	71	3	
trnD(guc)	1628	1699	H	72	8	
COX2	1708	2398	H	691	0	ATG/T
trnK(uuu)	2399	2472	H	74	1	
ATP8	2474	2650	H	177	-10	ATG/TAA
ATP6	2641	3323	H	683	0	ATG/TA
COX3	3324	4108	H	785	0	ATG/TA
trnG(ucc)	4109	4178	H	70	0	
ND3	4179	4527	H	349	0	ATG/T
trnR(ucg)	4528	4595	H	68	0	
ND4L	4596	4892	H	297	-7	ATG/TAA
ND4	4886	6266	H	1381	0	ATG/T
trnH(gug)	6267	6335	H	69	0	
trnS(gcu)	6336	6402	H	67	4	
trnL(uag)	6407	6479	H	73	0	
ND5	6480	8309	H	1830	-4	ATG/TAA
ND6	8306	8827	L	522	0	ATG/TAG
trnE(uuc)	8828	8896	L	69	4	
CYTB	8901	10041	H	1141	0	ATG/T
trnT(ugu)	10042	10115	H	74	-1	
trnP(ugg)	10115	10183	L	69	0	
D-loop	10184	10910		727	0	
trnF(gaa)	10911	10978	H	68	0	
12S	10979	11927	H	949	0	
trnV(uac)	11928	11998	H	71	1	
16S	12000	13693	H	1694	0	
trnL(uaa)	13694	13768	H	75	0	
ND1	13769	14743	H	975	6	ATG/TAA
trnI(gau)	14750	14819	H	70	-1	
trnQ(uug)	14819	14889	L	71	-1	
trnM(cau)	14889	14958	H	70	0	
ND2	14959	16005	H	1047	0	ATG/TAA
trnW(uca)	16006	16079	H	74	0	
trnA(ugc)	16080	16148	L	69	1	
trnN(guu)	16150	16222	L	73	40	
trnC(gca)	16263	16329	L	67	0	
trnY(gua)	16330	16397	L	68	1	

* Negative numbers indicate overlapping nucleotides between adjacent genes.

Table S8. Base composition of the *Leiognathus equula* mitochondrial genome

Regions	Size (bp)	T%	C%	A%	G%	AT (%)	AT skew	GC skew
Full genome	16398	24.2	30.1	30.7	15.0	54.9	0.117	-0.334
PCGs	11421	26.8	30.7	27.8	14.7	54.6	0.017	-0.353
tRNAs	1552	28.0	19.8	29.6	22.6	57.6	0.028	0.065
rRNAs	2643	18.1	27.5	34.8	19.6	52.9	0.316	-0.167
1st codon position	3807	20.4	27.5	28.6	23.5	49.0	0.166	-0.078
2nd codon position	3807	40.3	27.7	18.3	13.7	58.6	-0.376	-0.339
3rd codon position	3807	19.7	36.9	36.5	6.9	56.2	0.298	-0.685
12s	949	18.1	28.0	33.1	20.8	51.2	0.292	-0.149
16s	1694	18.1	27.2	35.7	19.0	53.8	0.328	-0.178
atp6	683	27.4	32.9	26.5	13.2	53.9	-0.016	-0.429
atp8	177	22.6	36.7	32.2	8.5	54.8	0.175	-0.625
cox1	1551	29.1	27.0	26.0	17.9	55.1	-0.056	-0.202
cox2	691	24.9	29.5	28.8	16.8	53.7	0.073	-0.275
cox3	785	26.1	30.7	27.5	15.7	53.6	0.026	-0.324
cytb	1141	27.1	31.6	28.6	12.7	55.7	0.027	-0.427
nad1	975	23.8	34.4	28.0	13.8	51.8	0.081	-0.426
nad2	1047	24.1	33.4	32.2	10.3	56.3	0.144	-0.528
nad3	349	30.4	33.2	23.2	13.2	53.6	-0.134	-0.432
nad4	1381	25.3	32.8	29.2	12.7	54.5	0.068	-0.439
nad4l	297	23.9	36.0	25.3	14.8	49.2	0.027	-0.417
nad5	1830	25.9	31.3	30.9	11.9	56.8	0.088	-0.451
nad6	522	42.0	11.5	10.9	35.6	52.9	-0.587	0.512
D-loop region	727	33.7	15.3	29.1	21.9	62.8	-0.070	0.173

Table S9. Codon number and relative synonymous codon usage (RSCU) of *L. equula* mitochondrial protein-coding genes

AA	Codon	Count	RSCU	AA	Codon	Count	RSCU
Phe	UUU(F)	95	0.85	Tyr	UAU(Y)	41	0.77
Phe	UUC(F)	129	1.15	Tyr	UAC(Y)	65	1.23
Leu	UUA(L)	82	0.77	Stop codon	UAA	5	2.86
Leu	UUG(L)	19	0.18	Stop codon	UAG	2	1.14
Leu	CUU(L)	112	1.05	His	CAU(H)	29	0.53
Leu	CUC(L)	154	1.44	His	CAC(H)	80	1.47
Leu	CUA(L)	238	2.23	Gln	CAA(Q)	85	1.75
Leu	CUG(L)	36	0.34	Gln	CAG(Q)	12	0.25
Ile	AUU(I)	129	0.84	Asn	AAU(N)	34	0.49
Ile	AUC(I)	178	1.16	Asn	AAC(N)	104	1.51
Met	AUA(M)	130	1.47	Lys	AAA(K)	74	1.8
Met	AUG(M)	47	0.53	Lys	AAG(K)	8	0.2
Val	GUU(V)	44	0.95	Asp	GAU(D)	19	0.58
Val	GUC(V)	51	1.1	Asp	GAC(D)	46	1.42
Val	GUA(V)	71	1.53	Glu	GAA(E)	76	1.65
Val	GUG(V)	20	0.43	Glu	GAG(E)	16	0.35
Ser	UCU(S)	36	0.82	Cys	UGU(C)	10	0.74
Ser	UCC(S)	74	1.69	Cys	UGC(C)	17	1.26
Ser	UCA(S)	72	1.65	Trp	UGA(W)	102	1.73
Ser	UCG(S)	13	0.3	Trp	UGG(W)	16	0.27
Pro	CCU(P)	41	0.72	Arg	CGU(R)	6	0.33
Pro	CCC(P)	97	1.7	Arg	CGC(R)	12	0.66
Pro	CCA(P)	85	1.49	Arg	CGA(R)	49	2.68
Pro	CCG(P)	5	0.09	Arg	CGG(R)	6	0.33
Thr	ACU(T)	60	0.76	Ser	AGU(S)	14	0.32
Thr	ACC(T)	131	1.66	Ser	AGC(S)	53	1.21
Thr	ACA(T)	118	1.49	Stop codon	AGA	0	0
Thr	ACG(T)	7	0.09	Stop codon	AGG	0	0
Ala	GCU(A)	53	0.67	Gly	GGU(G)	27	0.46
Ala	GCC(A)	144	1.82	Gly	GGC(G)	71	1.2
Ala	GCA(A)	110	1.39	Gly	GGA(G)	91	1.54
Ala	GCG(A)	9	0.11	Gly	GGG(G)	47	0.8
