# Supplementary material for: ﻿Morphometric and phylogenetic analysis of a commercial fish Leiognathusequula (Teleostei, Leiognathidae)
Source: Zookeys. 2024 Dec 4;1219:249–70. doi: 10.3897/zookeys.1219.130546 (PMC11635357; doi:10.3897/zookeys.1219.130546)
Supplement: Supplementary material 1 — Supplementary file [file zookeys-1219-249_article-130546__-s001.zip › Figure S1_on sale.pdf]

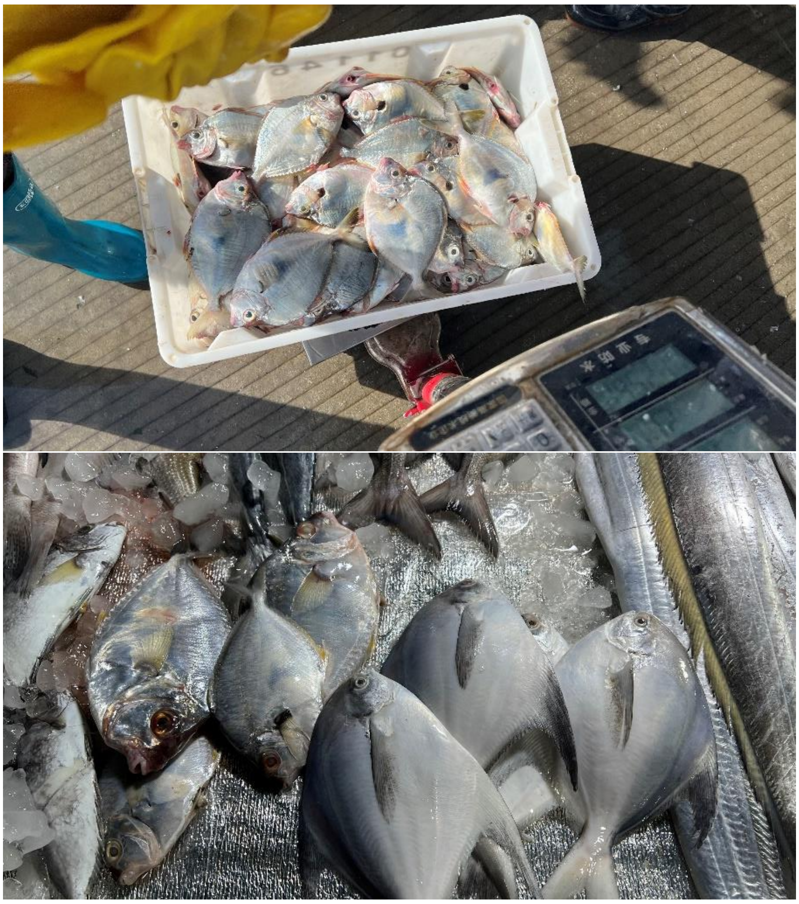

Figure S1. *Leiognathus equulus* was on sale at the landing ports (above) and fish markets (below) in Qinglan, Hainan, on 9 November 2023.
