# Supplementary material for: ﻿Morphometric and phylogenetic analysis of a commercial fish Leiognathusequula (Teleostei, Leiognathidae)
Source: Zookeys. 2024 Dec 4;1219:249–70. doi: 10.3897/zookeys.1219.130546 (PMC11635357; doi:10.3897/zookeys.1219.130546)
Supplement: Supplementary material 1 — Supplementary file [file zookeys-1219-249_article-130546__-s001.zip › Figure S2. Dorsal head.pdf]

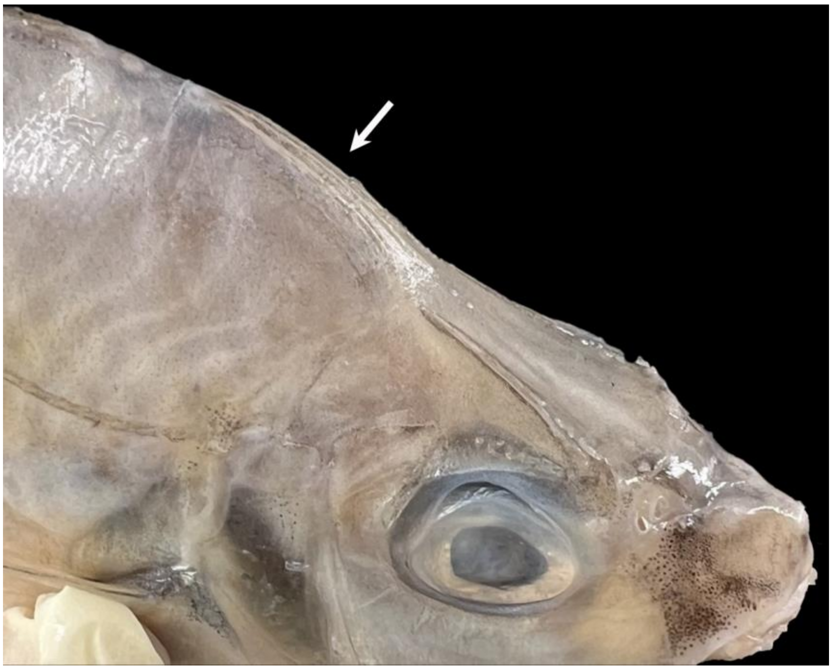

Figure S2. Dorsal head slightly triangular of *Leiognathus equulus*. DHS14327. Arrow indicates nuchal spine.
