# Supplementary figures and images for: ﻿Morphometric and phylogenetic analysis of a commercial fish Leiognathusequula (Teleostei, Leiognathidae)
Source: Zookeys. 2024 Dec 4;1219:249–70. doi: 10.3897/zookeys.1219.130546 (PMC11635357; doi:10.3897/zookeys.1219.130546)

16S\_BI

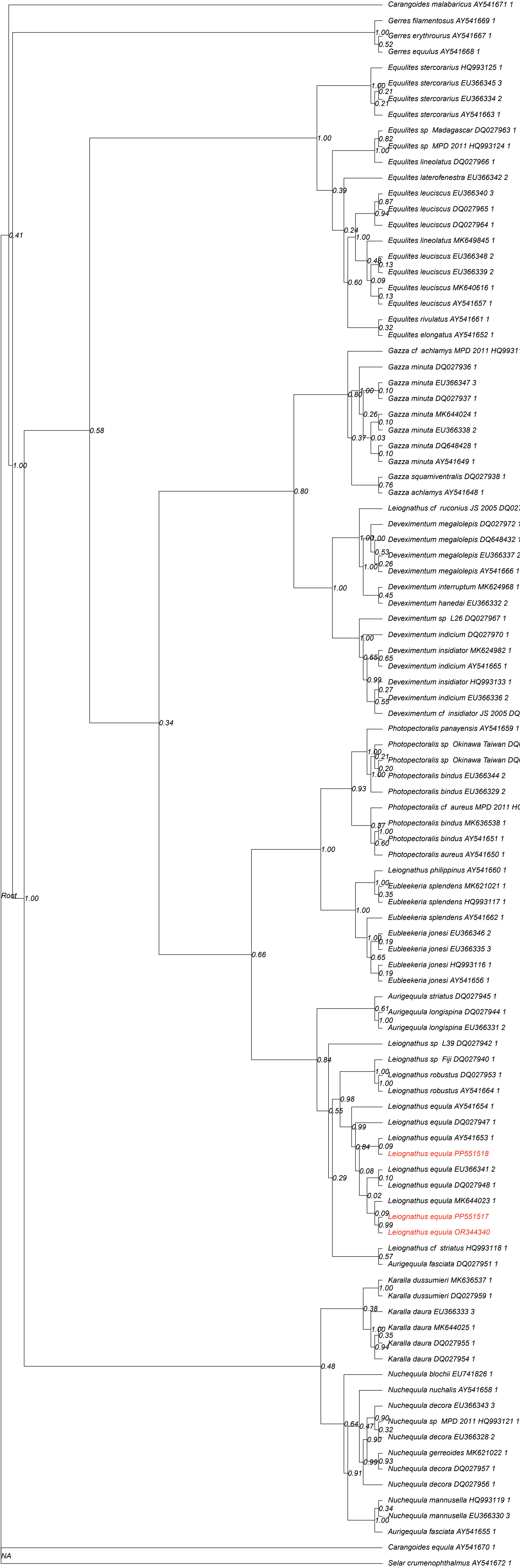

16S\_ML

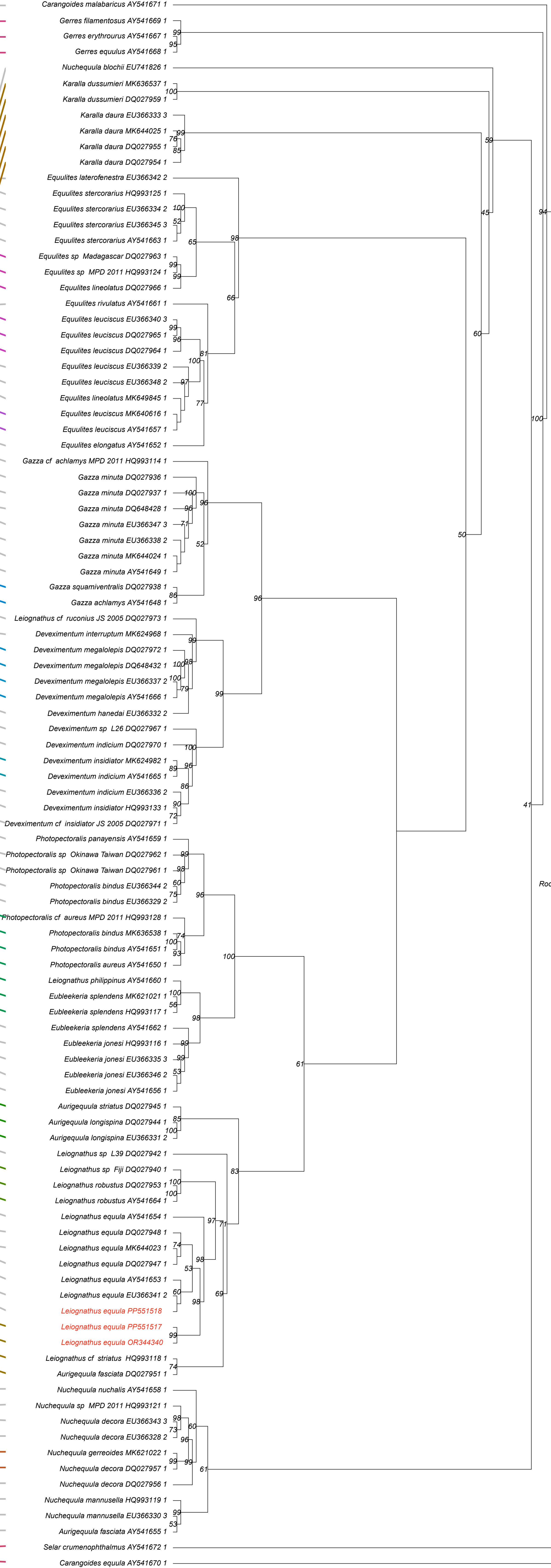

Supplement: Supplementary material 1 — Supplementary file [file zookeys-1219-249_article-130546__-s001.zip › Figure S3-16S.pdf]

COX1\_BI

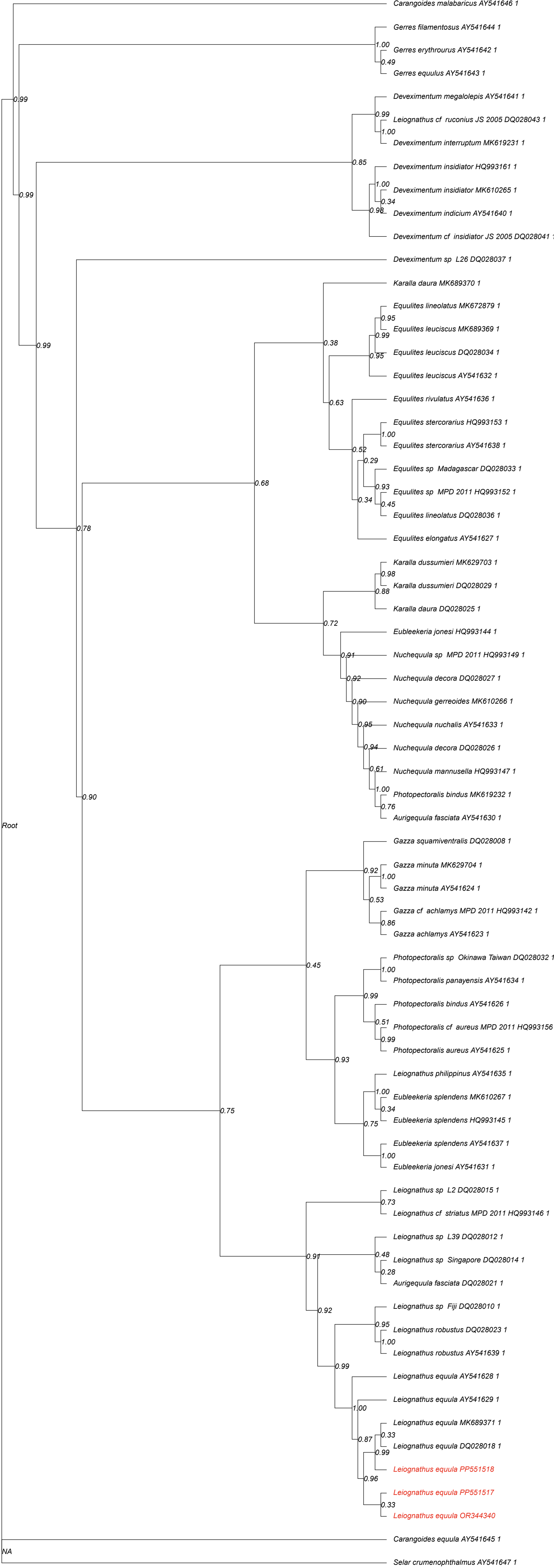

COX1\_ML

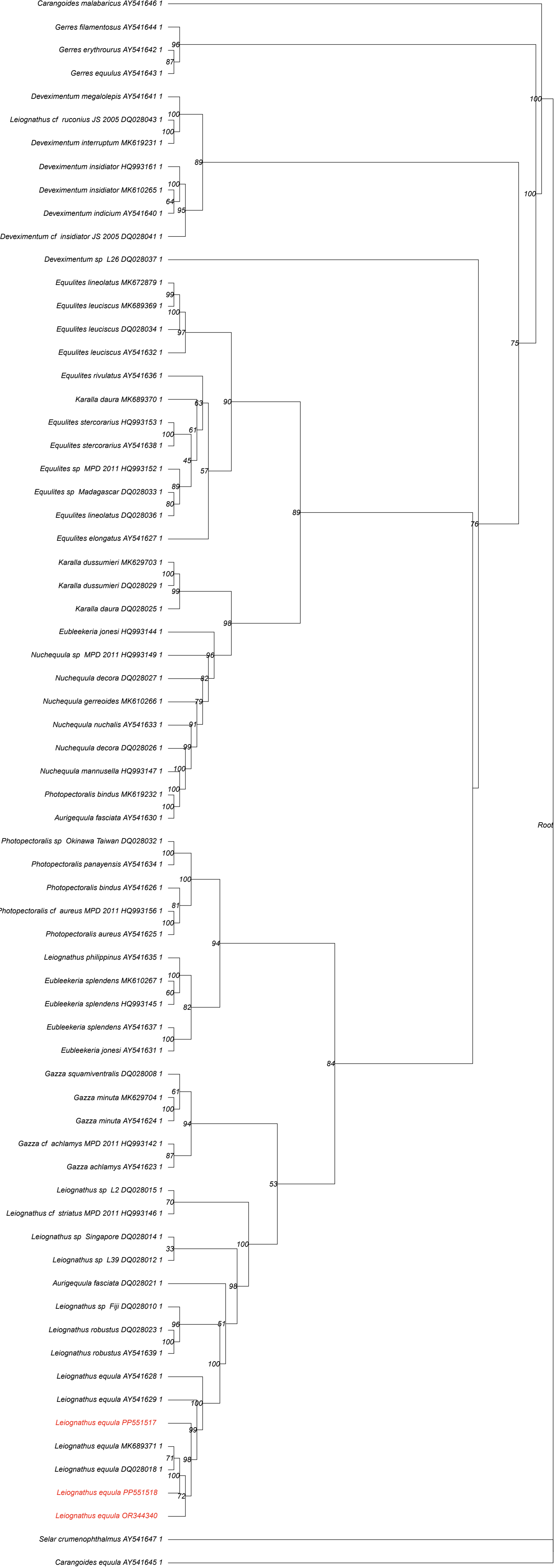

Supplement: Supplementary material 1 — Supplementary file [file zookeys-1219-249_article-130546__-s001.zip › Figure S4-COX1.pdf]

ND5\_BI

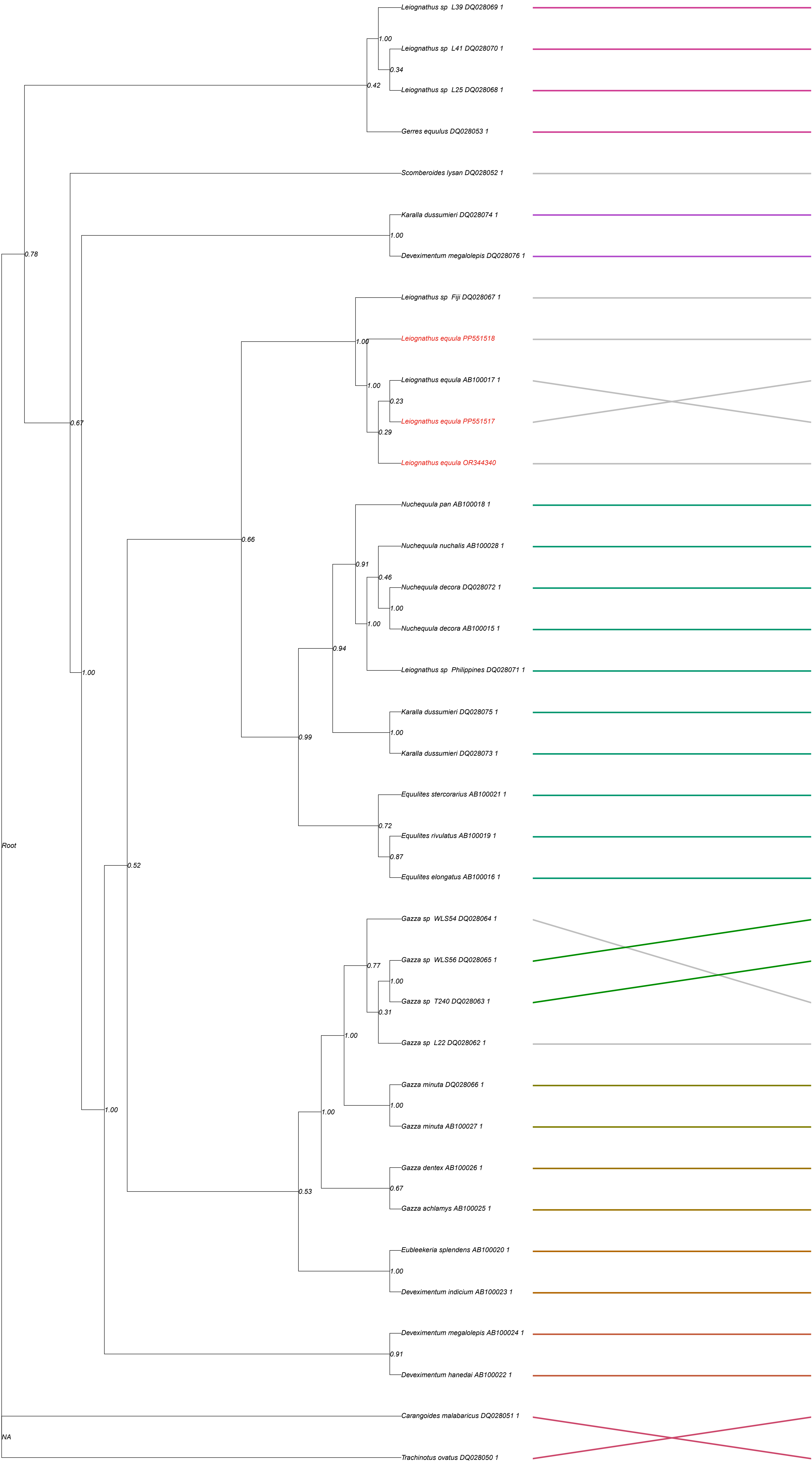

ND5\_ML

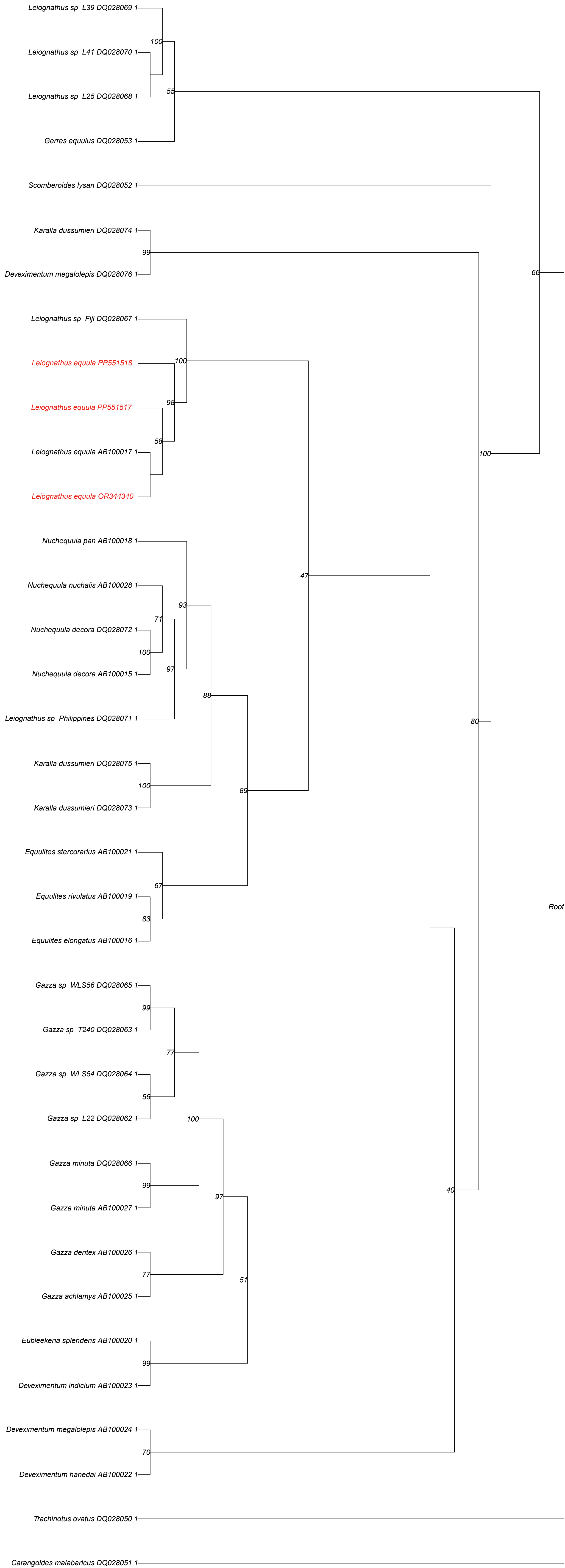

Supplement: Supplementary material 1 — Supplementary file [file zookeys-1219-249_article-130546__-s001.zip › Figure S5-ND5.pdf]
